# Not4-dependent targeting of *MMF1* mRNA to mitochondria limits its expression via ribosome pausing, Egd1 ubiquitination, Caf130, no-go-decay and autophagy

**DOI:** 10.1093/nar/gkad299

**Published:** 2023-04-24

**Authors:** Siyu Chen, George Allen, Olesya O Panasenko, Martine A Collart

**Affiliations:** Department of Microbiology and Molecular Medicine, Faculty of Medicine, University of Geneva, Institute of Genetics and Genomics of Geneva, Geneva, Switzerland; Department of Microbiology and Molecular Medicine, Faculty of Medicine, University of Geneva, Institute of Genetics and Genomics of Geneva, Geneva, Switzerland; Department of Microbiology and Molecular Medicine, Faculty of Medicine, University of Geneva, Institute of Genetics and Genomics of Geneva, Geneva, Switzerland; Department of Microbiology and Molecular Medicine, Faculty of Medicine, University of Geneva, Institute of Genetics and Genomics of Geneva, Geneva, Switzerland

## Abstract

The Ccr4–Not complex is a conserved multi protein complex with diverse roles in the mRNA life cycle. Recently we determined that the Not1 and Not4 subunits of Ccr4–Not inversely regulate mRNA solubility and thereby impact dynamics of co-translation events. One mRNA whose solubility is limited by Not4 is *MMF1* encoding a mitochondrial matrix protein. In this work we uncover a mechanism that limits *MMF1* overexpression and depends upon its co-translational targeting to the mitochondria. We have named this mechanism Mito-ENCay. This mechanism relies on Not4 promoting ribosome pausing during *MMF1* translation, and hence the co-translational docking of the *MMF1* mRNA to mitochondria via the mitochondrial targeting sequence of the Mmf1 nascent chain, the Egd1 chaperone, the Om14 mitochondrial outer membrane protein and the co-translational import machinery. Besides co-translational **Mito**chondrial targeting, Mito-ENCay depends upon **E**gd1 ubiquitination by **N**ot4, the **C**af130 subunit of the Ccr4–Not complex, the mitochondrial outer membrane protein **C**is1, **a**utophagy and no-go-dec**ay**.

## Graphical Abstract

**Graphical Abstract:**
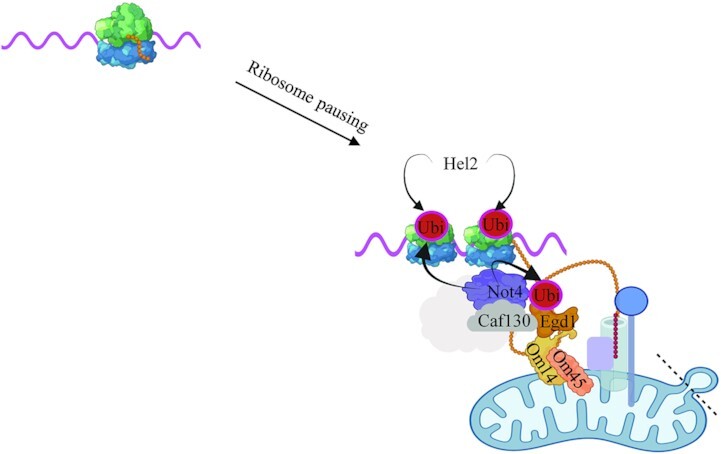
MitoENCay: ribosome pausing and mitochondrial targeting for co-translational degradation of excess mRNA[Reply By: AU]MitoENCay: ribosome pausing and mitochondrial targeting for co-translational degradation of excess mRNA.

## INTRODUCTION

Mitochondria are essential organelles with functions in cellular metabolism and homeostasis. They are of central importance for cellular energetics and participate in signaling mechanisms that ensure survival or promote death of cells under stress (1,2). Disruption of mitochondrial function has been associated with a large variety of diseases (3,4). Mitochondria have a characteristic architecture, delimited by outer and inner membranes, with inner membrane invaginations called cristae where oxidative phosphorylation occurs. The inner most aqueous compartment is the matrix. More than 1000 proteins have been identified in yeast mitochondria and nuclear genes encode over 99% of these. Hence, mitochondrial precursor proteins are for the most part produced in the cytoplasm and must be targeted to the appropriate mitochondrial compartments by targeting signals. In some cases the mitochondrial mRNAs are targeted to the mitochondria where they are translated and proteins co-translationally imported ((5–8) and for review see (9)), while in other cases proteins are synthesized in the cytosol and must reach the mitochondria post-translationally. Little is known about how such proteins reach the mitochondria *in vivo* (10). Targeting of the mRNAs to the mitochondria can be mediated by RNA binding proteins associating with 3’ untranslated regions (UTR) independently of translation, or by the mitochondrial targeting sequence of the nascent chains during translation. In budding yeast, the Puf3 RNA binding protein has important roles in targeting mitochondrial-specific mRNAs to the surface of mitochondria in respiratory conditions (6,7,11). For translation-dependent targeting, mitochondrial mRNAs can rely on the Nascent Polypeptide-associated Complex (NAC) chaperone, the Om14 or Sam37 mitochondrial outer membrane (MOM) proteins and Tom20 of the import machinery (8,12,13). The NAC chaperone is a heterodimer composed of alpha and beta subunits, respectively Egd2 and Egd1 or Btt1 in yeast, and it binds nascent peptides during translation (14,15). It is present in polysomes producing nuclear-encoded mitochondrial mRNAs (16,17). In all cases, the mitochondrial protein import machineries must take up the mitochondrial precursor proteins. These machineries are diverse and at least five major import pathways have been identified so far, each pathway characterized by a different machinery and different targeting signals (for review see (18)).

Important quality control (QC) systems respond to overexpressed mitochondrial precursors, to aberrant, mis-targeted or stalled nascent proteins at the MOM, to a saturated or compromised import channel, but also to excessive aggregated proteins in the cytoplasm, that all collaborate to maintain cellular homeostasis (for review see (19)). For instance, nascent chains stalled on the ribosome and engaged with mitochondrial import channels are rescued by the ribosome-associated quality control (RQC) complex, comprised of the Ltn1 ubiquitin ligase, the ATPase Cdc48, Rqc1 and Rqc2. RQC assembles on the 60S ribosomes containing unreleased peptidyl-tRNA. Vms1, a tRNA hydrolase that releases the stalled polypeptide chains engaged by the RQC (20), antagonizes Rqc2 to prevent elongation of the nascent chain with carboxy-terminal alanyl/threonyl (CAT) tails. The Hel2 ubiquitin ligase is a subunit of the ribosome-associated quality control trigger complex (RQT) and is essential to trigger RQC (21). Another example is ‘MitoCPR’, a response that facilitates degradation in the cytosol of unimported mitochondrial precursor proteins accumulating at the mitochondrial translocase. It involves inducing expression of Cis1 at the translocase, that functions with the AAA^+^ adenosine triphosphatase Msp1 and the proteasome (22). This improves mitochondrial import during import stress. ‘MAD’ is the response by which the components of the ubiquitin proteasome system (UPS) are recruited to the MOM to trigger degradation of proteins peripherally associated with the MOM, integral MOM proteins, mitochondrial intermembrane space proteins, and potentially also inner membrane or matrix proteins (23). An increase of mitochondrial precursor proteins in the cytosol triggers the ‘UPR^am^’, leading to increased proteasome assembly by the enhanced activity of the proteasome assembly factors Irc25 and Poc4, that degrades excess proteins (24). Inversely, upon accumulation of high levels of aggregated proteins in the cytoplasm, Hsp104 helps to dissociate the aggregates. Thereby it contributes to MAGIC (Mitochondria As Guardian In Cytosol), a mechanism by which aggregation-prone proteins can enter via import channels the mitochondrial intermembrane space or matrix for degradation (25). All of these mechanisms are indicative of a major cross-talk between the cytoplasm and the mitochondrion to maintain protein homeostasis. In addition to these mechanisms, autophagy can sequester and remove unnecessary or dysfunctional components in bulk from the cytoplasm and mitophagy is the specific form of autophagy that serves to remove damaged mitochondria (for review, see (26)).

Ccr4–Not is a conserved, multi-subunit complex that plays multiple roles in the control of gene expression and mRNA metabolism. In yeast Ccr4–Not consists of 9 subunits: Ccr4, Caf1, Caf40, Caf130 and the five Not proteins (Not1, Not2, Not3, Not4 and Not5) (27–30). Our current knowledge about the functional roles of this complex is that its regulatory functions span the entire lifespan of mRNAs, from their synthesis to their decay. Moreover, it plays extensive roles in translation and protein turnover (31–33). Recent studies have uncovered key roles of the Not proteins in co-translational processes, such as co-translational assembly of proteins (32,34,35) and translation elongation dynamics (36). Not5 can associate with the E site of post-translocation ribosomes bearing an empty A site. This has been proposed to enable the Ccr4–Not complex to monitor the translating ribosome for mRNA turnover according to codon optimality (37). Consistently, depletion of Not5 changes A-site ribosome dwelling occupancies inversely to codon optimality (36). In addition, ubiquitination of Rps7A by Not4 can contribute to degradation of mRNAs by no-go-decay (NGD) in conditions where the RQC response is defective (38).

Recently, we noted that Not1 and Not4 depletions inversely modulated mRNA solubility thereby determining dynamics of co-translation events (39). Notably, mRNAs encoding mitochondrial proteins were enriched amongst mRNAs whose solubility was most extremely inversely regulated upon Not1 and Not4 depletion. In this context, it is interesting to note that the Ccr4–Not complex interacts with factors that contribute to targeting of mitochondrial mRNAs to the mitochondria: Egd1 is ubiquitinated by Not4 (40) and Puf3 recruits the Ccr4–Not complex to its target mRNAs for degradation (41–44). Moreover, mitochondrial mRNAs are enriched amongst mRNAs bound by Not1 in a Not5-dependent manner (33).

In our current study we uncover an integrated QC mechanism that limits levels of a mitochondrial mRNA co-translationally and mobilizes components of several of the QC systems linking cytoplasm and mitochondria described above, as well as Ccr4–Not subunits. We focused our attention on one nuclear-encoded mitochondrial mRNA, *MMF1*, more soluble upon Not4 depletion (39). *MMF1* encodes a mitochondrial matrix protein required for transamination of isoleucine and it couples amino acid metabolism to mitochondrial DNA maintenance (45). It forms a homotrimer proposed to interact with a trimer of Mam33 (46), a translational activator in yeast mitochondria (47). We determine that Not4 limits Mmf1 overexpression during fermentative growth by contributing to ribosome pausing and promoting the co-translational docking of its mRNA to mitochondria via the mitochondrial targeting sequence of the Mmf1 nascent chain, Egd1 and the co-translational import machinery. Accumulation of excessive *MMF1* mRNA, Mmf1 precursor and mature Mmf1 protein is then avoided in a mechanism requiring Egd1 and Rps7 ubiquitination by Not4, Caf130, Cis1, RQC and NGD, Hsp104, as well as autophagy, a mechanism that we have called Mito-ENCay.

## MATERIALS AND METHODS

### Yeast strains and plasmids

The strains, oligos, plasmids and antibodies used in this study are listed in Supplementary Table S1. Yeast strains were grown in rich medium with 2% glucose (YPD) or in synthetic drop out medium selective for plasmid maintenance. For copper induction, cells were grown to exponential phase after dilution of an overnight culture to OD_600_ of 0.3 and a stock solution of 0.1 M CuS0_4_ was added to a final concentration of 0.1 mM. To arrest protein synthesis a stock solution of cycloheximide (CHX) was added to a final concentration of 0.1 mg/ml in the growth medium.

The reporter plasmid expressing Mmf1 fused to Flag (pMAC1211) was constructed by cloning a PCR fragment amplified with oligos 935 and 936 and genomic DNA, digested by MfeI and Not1 in pE617 digested by EcoRI and Not1. The reporter plasmid expressing Mmf1 without the MTS (pMAC1327) was made using PCR with oligos 1028 and 1030, transformation of the PCR fragment with pE617 digested by EcoRI and Not1 into yeast, and plasmid rescue. The one with the Cox4 MTS (pMAC1328) was made similarly with oligos 1029 and 1030. The one expressing Cox4 (pMAC1200) was made similarly with oligos 691 and 692, except that the PCR fragment was digested by EcoRI and Not1. MS2 loops were added in the pMAC1211 and pMAC1327 plasmids by co-transforming into yeast the pMAC1211 and pMAC1327 plasmids digested with SacI and a PCR fragment obtained with oligos 1087 and 1088 and pE659, leading to pMAC1365 and pMAC1367. Constructs were verified by sequencing with oligo 1113. For both plasmids the *URA3* marker was swapped to the *HIS3* marker by transforming pE23 digested by SmaI and selection of His + Ura- colonies, followed by plasmid rescue leading to plasmids pMAC1430, and pMAC1431. For pMAC1211, the *URA3* marker was swapped to the *LEU2* marker by transforming pE24 digested by StuI and selection of the Leu + Ura- colonies followed by plasmid rescue leading to pMAC1342. The plasmid with altered codons around the ribosome-pause-site on *MMF1*, pMAC1425, and the plasmid with the *MMF1*-MTS replacement in *COX4*, pMAC1424, were created by a Q5 Site-Directed Mutagenesis Kit (NEB, E0554S) and oligo pairs 1301,1302 and 1297 with 1298. All plasmids were verified by sequencing. Plasmids encoding Egd1, Not4 and Rli1 derivatives have already been published (see Supplementary Table S1).

### Protein ubiquitination assay

This method was done as previously described (40). A plasmid expressing 6His-tagged ubiquitin under the control of the inducible *CUP1* promoter was transformed into cells. The transformants were cultured in medium selective for plasmid maintenance in the presence of 0.1 mM CuSO_4_. 100 OD_600_ of cells were harvested when they reached late exponential phase. Cell pellets were weighed and resuspended with G-buffer (100 mM sodium Pi, pH 8.0, 10 mM Tris–HCl, 6 M guanidium chloride, 5 mM imidazole, 0.1% Triton X-100) at 100 mg/ml. 0.6 ml of glass beads was added and cells were disrupted by bead beating for 15 min at room temperature (RT). Following centrifugation, 20 μl of the supernatant was taken as total extract (TE), and 700 μl of the supernatant was mixed with 30 μl of nickel-nitrilotriacetic acid-agarose (Ni-NTA, Qiagen) for 2 h at RT with mild rotation. U-buffer (100 mM sodium Pi, pH 6.8, 10 mM Tris–HCl, 8 M urea, 0.1% Triton X-100) was used to abundantly wash the Ni-NTA-agarose to which ubiquitinated proteins were bound. SB was added directly to the Ni-NTA with the ubiquitinated proteins for analysis by western bloting with relevant antibodies.

### Confocal microscopy

Cells were grown in 2% glucose synthetic medium and harvested at an OD_600_ between 0.6 and 1.2. Two OD_600_ of cells were then spun at 3000 g for 5 min at RT, washed and fixed with 600 μl of 4% paraformaldehyde for 30 min at RT. Fixed cell pellets were resuspended in 200 μl of PBS, and 10 μl were evenly distributed on polysine coated slides. Nail polish was used to mount the coverslips, and images of the prepared slides were acquired using a stand confocal microscope (LSM800 Airyscan) with a 63× oil objective (NA = 1.4). Z-stacking was employed to acquire each image at fixed intervals of 0.23 μm. To determine the shortest distance between mRNAs and mitochondria, the Imaris software (version 9) was used, with its sport model generating the 3D model of mRNA and its surface model building the 3D model of mitochondria. Statistical analysis was performed using Prism9, with a two-tailed unpaired *t*-test, with Welch's correction. Each sample was assessed for >100 spots.

### Protein extracts, SDS- or native PAGE and western blotting

Total protein extracts were prepared by incubating pelleted yeast cells in 0.1 M NaOH for 10 min at RT. After a quick spin in a microfuge, the cell pellet was resuspended in 2 X sample buffer (post-alkaline lysis). Samples were subjected to SDS-PAGE and western blotting according to standard procedures. For native gels (48), ready-made native 3–12% Bis–Tris gels were used (Invitrogen) according to instructions. Briefly, 20 OD_600_ of cells were harvested at exponential growth. Cells were disrupted by 0.2 ml glass beads in the presence of 0.4 ml lysis buffer (20 mM HEPES pH 7.5, 20 mM KCl, 10 mM MgCl_2_, 1% Triton X-100, 1 mM DTT, 1 mM PMSF, supplemented with a cocktail of protease inhibitors (Roche)). The indicated amount of total protein extract was mixed with native sample buffer from Invitrogen. Following the electrophoresis (150 V, 3 h, 4°C) and transfer (40 W, 1 h, RT) to PVDF or nitrocellulose membranes, the blots were incubated with the indicated antibodies. To quantify expression of reporter proteins, after revelation of the western blots the captured images were imported into the Fiji software and converted to an 8-bit format. Background subtraction was performed before image analysis. The processed images were measured, and the pixel values over the Flag signal were normalized over the pixel values for the Egd2 signal.

### RNA preparation and analysis

RNA extraction and analysis was performed as previously described (33). Relative mRNA abundances were determined by RT-qPCR with the Pfaffl method (49). For normalization, we measured *EGD2* as an invariable control mRNA and calculated the ΔCT values. Oligos 687 and 999 were used for *MMF1* reporter mRNAs, 714 and 999 for *COX4* reporter mRNA and oligos 1000 with 1001 for *EGD2*.

### Fractionation and mitochondria isolation

Mitochondrial fractionation was performed from 1 l of yeast cells as previously published (50) and simplified (http://www.jove.com/details.php?id=1417) with minor modifications (Tris–HCl rather than Tris/H_2_SO_4_ and addition of protease inhibitors). Briefly, cell pellets were washed with a buffer containing 100 mM Tris–HCl (pH 9.4) and 10 mM DTT. Spheroplasts were then generated using Zymolyase 100T (Biological, Z1004, US). A homogenization buffer consisting of 0.6 M sorbitol, 10 mM Tris–HCl (pH 7.4), 1 mM EDTA, 1 mM PMSF and 0.2% BSA was supplemented with a protease inhibitor cocktail (Roche). The cells were broken using a glass dounce homogenizer, and 20 μl of the lysate was collected as input. The remaining lysate was spun at 1500 g for 5 min at 4°C to remove cell debris and nuclei. The supernatant was then spun at 3000 g for 5 min at 4°C, and mitochondria were pelleted from this next supernatant at 12 000 g for 15 min at 4°C. The remaining supernatant fraction was collected as ‘cytoplasm fraction’. The pellet was washed with SEM buffer (250 mM sucrose, 1 mM EDTA, 10 mM Mops, pH 7.2) and resuspended in SEM buffer for loading onto a 3-step sucrose gradient. After centrifugation at 134 000 g in a Beckman SW41 TI swinging-bucket rotor for 1 h at 2°C, the purified mitochondria were recovered from the 60% to 32% interface. The collected mitochondrial fraction was further pelleted by centrifugation at 10 000 g at 2°C and resuspended in a storage buffer (SEM buffer without sucrose). Input, cytoplasm and mitochondrial fractions were mixed with sample buffer for western blot analysis.

### Ribosome profiling and bioinformatic analysis

Samples for ribosome profiling were prepared and analyzed previously (35) and the data was extracted to show ribosome footprints on *MMF1* and *COX4* mRNAs in wild type and *not4Δ*.

### RNA-seq and solubility analyses

The data was generated and analyzed in (39). We extracted the data to show the change in solubility of *MMF1* and *COX4* upon Not1 and Not4 depletion.

## RESULTS

### Mmf1, but not Cox4, is co-translationally imported

To start dissecting how the Ccr4–Not complex regulates solubility of mRNAs to regulate co-translation events, we focused our attention on two mitochondrial mRNAs, *MMF1* and *COX4*, rendered more soluble upon Not4 depletion, but less soluble upon Not1 depletion (Supplementary Figure S1A) (39). Both mRNAs express mitochondrial precursor proteins with an N-terminal cleavable targeting sequence and assemble into multi-protein complexes. Cox4 is a component of the respiratory complex IV located in the mitochondrial inner membrane (51) whereas the Mmf1 trimer resides in the matrix (45).

**Figure 1.**
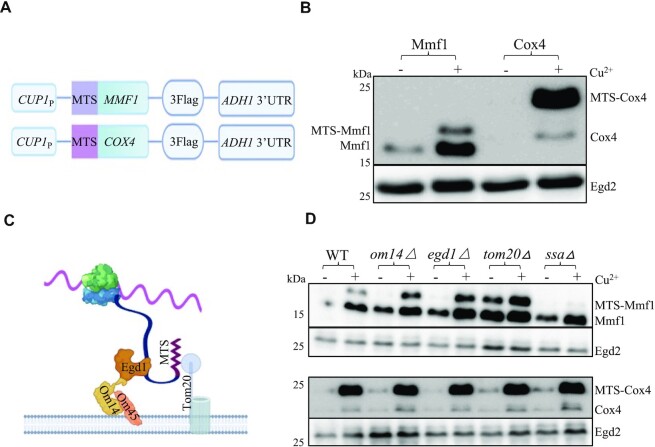
Mmf1 but not Cox4 is co-translationally imported and regulated. (**A**) Cartoon of the reporter constructs used in which coding sequences are fused to a C-terminal Flag tag, under the control of the *CUP1* inducible promoter. (**B**) Wild type cells (WT) transformed with the reporters and growing exponentially in medium selective for the plasmids were untreated (-) or treated (+) with 0.1 mM CuS0_4_ (Cu^2+^) for 10 min. Cells were collected for total protein analysis by western blotting with antibodies to Flag or with antibodies to Egd2 to control for protein loading. Precursor and mature Mmf1 and Cox4 are indicated respectively left and right of the blot. Molecular weight markers are indicated on the left. (**C**) Cartoon of the co-translational import machinery with the nascent chain exposed from the ribosome interacting with the Egd1 chaperone itself docking onto the Om14 MOM protein interacting with Om45, and the MTS of the nascent chain recognizing Tom20 to enable transfer of the nascent chain into the Tom channel. (**D**) Analysis of the reporters as in panel B in the indicated strains (upper panels Mmf1 and Egd2, lower panels Cox4 and Egd2).

To study the regulation of *MMF1* and *COX4* expression dependent upon their coding sequences, we used reporter constructs with the heterologous and inducible *CUP1* promoter and the heterologous *ADH1* 3’UTR in between which we cloned the *MMF1* and *COX4* coding sequences (CDS) fused to a C-terminal Flag tag (Figure 1A). We transformed the plasmids in wild type cells and tested expression of the reporter before and after induction with copper for 10 min. Before induction some mature Mmf1 was already detectable, due to some leakage of the *CUP1* promoter. Immediately after induction, levels of unprocessed and mostly mature Mmf1 were increased, whilst mostly unprocessed Cox4 was visible, with very low levels of mature protein (Figure 1B). This suggests that processing of induced Mmf1 might be faster than that of Cox4, compatible with the idea that the former but not the latter might be co-translationally processed and imported.

To look at this further, we transformed the two plasmids in strains defective for the mitochondrial co-translational import machinery (8), namely cells lacking the Egd1 chaperone or its receptor on the MOM, Om14 or finally the Tom20 receptor (see cartoon on Figure 1C). We also transformed the plasmids in cells defective for the cytoplasmic Hsp70 chaperones (called Ssa1-4 in yeast) reported to contribute to effective post-translational import of mitochondrial proteins (52). As before, we analyzed the expression from the reporter plasmids before and after a 10 min induction with copper. After induction, we noted elevated levels of the unprocessed Mmf1 protein in the mutants of the co-translational machinery but not in the *ssaΔ* mutant (Figure 1D, upper panels). The ratio of unprocessed to mature Mmf1 was also increased in all mutants relative to wild type, except for the *ssaΔ* mutant (Supplementary Figure S1B). In all strains, very little Cox4 was detectable before induction and after induction we noted mostly unprocessed Cox4, at levels similar in all strains (Figure 1D, lower panels). Since the *MMF1* and *COX4* reporters have identical 5’ and 3’ untranslated sequences, the difference in their regulation must depend upon the coding sequences. Hence, these results are compatible with a negative regulatory role of the co-translational import pathway for control of the expression of *MMF1*, but not *COX4*, coding sequences.

**Figure 2.**
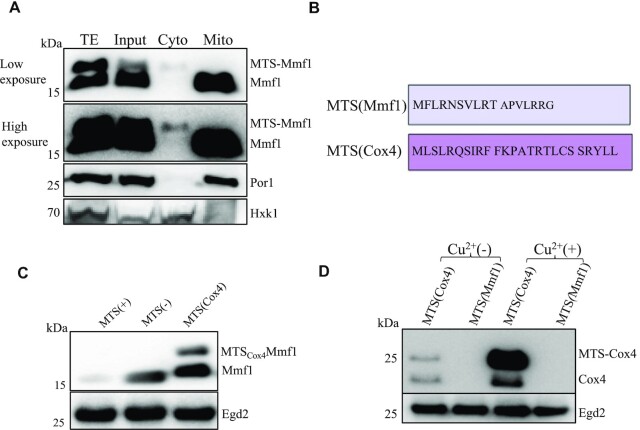
The *MMF1* MTS represses expression and can exert this effect on a heterologous gene. (**A**) *egd1Δ* cells expressing the Mmf1 reporter were lysed after a 10 min copper induction for a purification of mitochondria. Total extracts (TE), cell lysate (Input), cytosolic fraction (Cyto) or the mitochondrial fraction (Mito) were tested by western blotting for expression of Mmf1 with antibodies to Flag. A low and high exposure are shown. Antibodies to a mitochondrial protein Por1 or a cytosolic protein hexokinase (Hxk1) were used as a control for the fractionation procedure. (**B**) Amino acid sequence of the Mmf1 and Cox4 MTS sequences. (**C**) Expression of the *MMF1* reporter with MTS (left), without MTS (middle), or with the Cox4 MTS instead of its own MTS (right), in wild type cells growing exponentially analyzed by western blotting as in Figure 1B. (**D**) Expression of the *COX4* reporter with its MTS or with the *MMF1* MTS (as indicated) before and after copper induction analyzed as in panel (C).

By performing an 18 h cycloheximide (CHX) chase after copper induction for some of the same strains tested above, and additionally cells lacking the Om14 partner Om45, we noted again that after induction the Mmf1 precursor was overexpressed except in *ssaΔ* cells. Furthermore, Mmf1, whether precursor or mature, was relatively stable. Instead, the overexpressed Cox4 turned over rapidly and neither precursor nor much mature protein was detectable already by 2 h of CHX chase in all strains tested (Supplementary Figure S1C). We considered the possibility that the difference in stability of the Mmf1 and Cox4 reporter proteins could be related to the Cox4 precursor being cytoplasmic, while the Mmf1 precursor instead could be ‘stuck’ in the mitochondrial import machinery. We thus prepared mitochondria and cytoplasmic fractions from *egd1Δ* cells after copper induction that have high levels of both precursor and mature Mmf1 reporter protein. We followed on one hand the Mmf1 reporter protein with antibodies to Flag, and as a control for the fractionation procedure, we evaluated the presence of the mitochondrial Por1 protein (53) and the Hxk1 hexokinase (54) for the cytoplasmic fraction, with specific antibodies. The Mmf1 precursor was unstable in cell extracts, but nevertheless it was detected in the cytoplasmic fraction but not in the mitochondrial fraction, whereas like Por1, the mature Mmf1 was detectable exclusively in the mitochondrial fraction (Figure 2A).

Both Mmf1 and Cox4 have an N-terminal cleavable MTS sequence, but the amino acid composition of the MTS is very different (Figure 2B). We investigated the role of the mitochondrial targeting sequence (MTS) for regulation of Mmf1 expression, and the ability of the Cox4 MTS to replace the Mmf1 MTS. Mmf1 without its MTS or with the Cox4 MTS to replace its own MTS, was overexpressed (Figure 2C). These results indicate that the Mmf1 MTS is necessary to limit Mmf1 expression, and that the Cox4 and Mmf1 MTS are not functionally interchangeable for this function. We then replaced the MTS of Cox4 by that of Mmf1 and tested expression before and after copper induction. The replacement of the Cox4 MTS by that of Mmf1 totally repressed expression of Cox4 (Figure 2D). These results demonstrate that specifically the MTS of Mmf1 represses expression of the reporter protein to which it is fused.

### Regulation of *MMF1* but not *COX4* expression requires Not4 and the MTS

**Figure 3.**
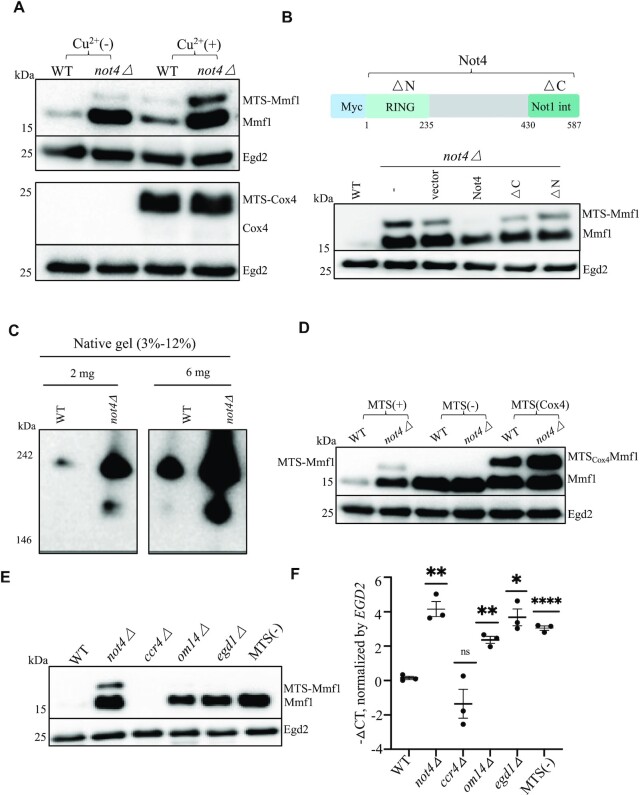
Overexpression of *MMF1* mRNA and protein in cells lacking Not4 or when Mmf1 lacks its MTS is epistatic. (**A**) Analysis of the reporters was evaluated in wild type and *not4Δ* cells as in Figure 1B. (**B**) Top: cartoon of the Myc6-Not4 coding sequence. The RING domain is located before amino acid 235 and the Not1-interaction domain is located after amino acid 430. Bottom: wild type cells (WT), *not4Δ* cells (–) or *not4Δ* cells transformed with plasmids expressing with an N-terminal Myc tag, wild type Not4, a derivative lacking the RING domain (ΔN) or a derivative lacking the Not1-interacting C-terminal domain (ΔC) and the *MMF1* reporter, were analyzed before copper induction as in panel A. (**C**) The indicated amounts of total soluble protein extract from wild type or *not4Δ* cells expressing TAP-tagged Mmf1 (the TAP tag has a calmodulin-binding entity and a Protein A entity) from its endogenous locus were analyzed by Native PAGE and western blotting with PAP antibodies. (**D**) Wild type and *not4Δ* cells were analyzed for expression of the *MMF1* reporter without the MTS or with the *COX4* MTS before copper induction as in panel A. (E and F) Wild type and the indicated mutant cells transformed with the *MMF1* reporter or wild type cells transformed with the *MMF1* reporter without the MTS as indicated were collected at the exponential growth phase without copper induction and analyzed by western blotting with antibodies to Flag (**E**) and by RT-qPCR (**F**). The *EGD2* protein and mRNA were used as a control for loading. The *MMF1* reporter mRNA levels were plotted to show means ± SD of –ΔCT values. The level of significant change, relative to WT is indicated with asterisks using a two-sided, Welch, unpaired *t*-test (*n* = 3).

We next tested expression of the Mmf1 and Cox4 reporters before and after copper induction in cells lacking Not4, because of Not4’s role in the regulation of the solubility of the *MMF1* and *COX4* mRNAs mentioned above (39). The expression of the Mmf1 precursor and mature protein was much higher in *not4Δ* (Figure 3A, upper panels). In contrast the expression of Cox4 was mostly indistinguishable between the wild type and mutant (Figure 3A, lower panels). Since the overexpression of the Mmf1 reporter was already detectable in mutant cells without copper induction due to leakage of the *CUP1* promoter, we worked further without copper induction.

We tested which functional domains of Not4 were important for control of Mmf1 expression and transformed *not4Δ* cells carrying the reporters with plasmids encoding wild type or mutant Not4 derivatives, in particular Not4 mutants lacking their C-terminal Not1-interaction domain or the N-terminal RING domain (55). Only wild type Not4 showed complementation of the Mmf1 overexpression. Notably however, the complementation from the plasmid expressing wild type Not4 was only partial (Figure 3B), maybe because of the presence of an N-terminal tag, or because Not4 is expressed from an episome rather than from the genomic locus.

The Not proteins are known to be important for co-translational assembly of specific protein complexes (34,35). Mmf1 forms homotrimers proposed to assemble with Mam33 trimers (46). We thus questioned whether Mmf1 complexes were appropriately formed in cells lacking Not4 and analyzed extracts of wild type and mutant cells expressing endogenous tagged Mmf1 expressed from its endogenous locus on native gels. Mmf1 from all strains migrated with a size between 146 and 242 kDa, larger than expected for Mmf1 homotrimers. Hence, the same apparent Mmf1 complexes could be formed in wild type cells and cells lacking Not4. However, faster migrating Mmf1 complexes were additionally seen in cells lacking Not4 (Figure 3C). These faster migrating Mmf1 complexes likely reflect higher expression levels of Mmf1 compared to its partner proteins, though we cannot exclude that they indicate ineffective complex assembly in mutant cells if the partner proteins are not limiting.

We next questioned whether increased expression of Mmf1 due to the absence of Not4 and the MTS were additive. However, the expression of Mmf1 without its MTS or with the Cox4 MTS was not further increased in *not4Δ* (Figure 3D). Hence Not4 and the Mmf1 MTS are epistatic with regard to their regulation of the Mmf1 reporter.

As mentioned above, overexpressed Cox4 turns over rapidly, whilst Mmf1 is stable, leaving open the possibility that the overexpression of Mmf1 but not Cox4 in mutants could be explained by this differential protein turnover. In such a case, we would not expect a change in *MMF1* mRNA levels. However, when Mmf1 protein was overexpressed in the different mutants or when Mmf1 was expressed without its MTS (Figure 3E), the *MMF1* mRNA was also overexpressed (Figure 3F). Instead, the levels of the *COX4* reporter mRNA were unaffected in all mutants, except in *not4Δ* (Supplementary Figure S2A). We also noted a very striking elevation of the *MMF1* but not *COX4* reporter mRNA in the *tom20Δ* mutant (Supplementary Figure S2B). These results indicate that the MTS and co-translational import machinery have a negative regulatory effect on the mRNA of the *MMF1* reporter. Interestingly, not only the Cox4 reporter protein levels (Figure 2D) but also the *COX4* reporter mRNA levels were reduced by the replacement of the Cox4 MTS by the Mmf1 MTS (Supplementary Figure S2C). Thus, the negative regulatory effect of the *MMF1* MTS can be transferred to heterologous coding sequences. Interestingly, neither reporter mRNA was affected in cells lacking the Ccr4 deadenylase subunit of the Ccr4–Not complex (Figure 3F and Supplementary Figure S2A). On the other hand, both Cox4 and Mmf1 proteins were increased in mutants of the proteasome, but in this case the mRNA levels were not significantly changed (Supplementary Figure S2D and E).

Taken together, these results show that repression of the *MMF1* but not *COX4* reporter is exerted at the mRNA level, by the Mmf1 MTS and components of the co-translational import machinery as well as by Not4, but independent of the Ccr4 deadenylase. This supports a model in which repression of *MMF1* overexpression occurs co-translationally at the mitochondrial co-translational import machinery.

### Ribosome pausing defined by codon context and Not4 regulate Mmf1 expression

**Figure 4.**
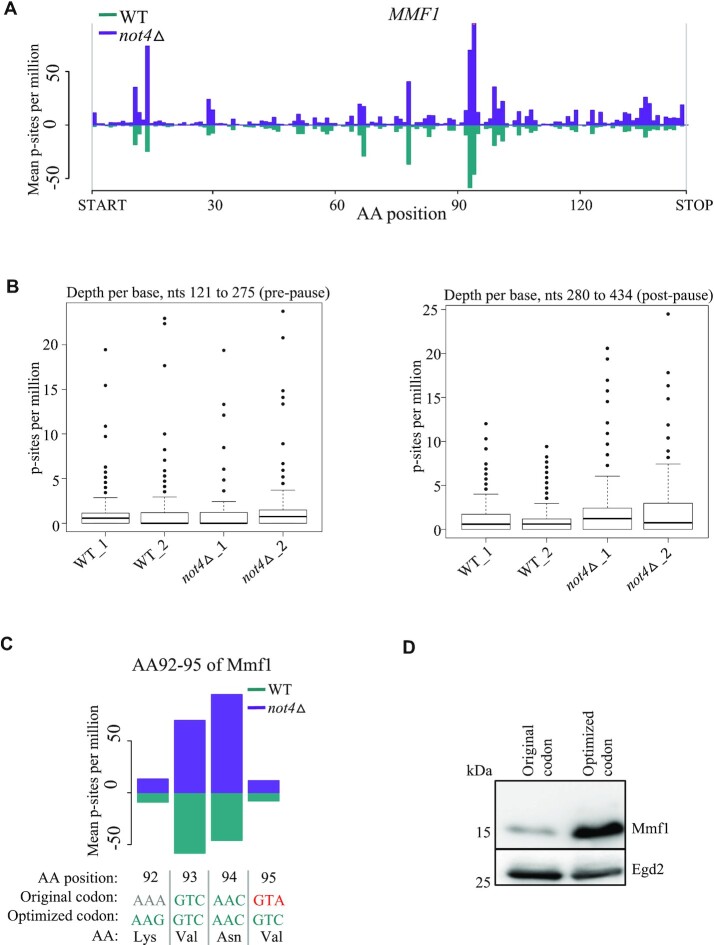
Translation dynamics according to codon optimality contributes to regulate Mmf1 expression. (**A**) Profiles of ribosome footprints (P-site depth plots) on *MMF1* with footprints in wild type cells in green and those in *not4Δ* cells in purple. The number of P-sites, per million genome-wide for each sample, covering each CDS codon with corresponding amino acid position indicated (AA position) is calculated, averaged for each condition and plotted. (**B**) Quantification of mRNA footprints in wild type and *not4Δ* cells for duplicate samples on equal segments of the mRNA before (left) and after (right) the apparent ribosome pausing site. Boxplots of P-sites per million for each base of the *MMF1* CDS in WT and *not4Δ* cells for the region between the large pause and the stop codon (nucleotides 280–434, right panel) and an equally-sized region just upstream of the pause (nucleotides 121–275, left panel). Only the region post-pause shows significant changes (DESeq2 p-value = 3.19e-5). (**C**) Visualization of the 4 codons at the *MMF1* ribosome pause site with encoded amino acids. Codons in blue are amongst the 15 most optimal and in red amongst the 15 most non-optimal yeast codons. The first line indicates the position, the second line indicates the codon in the wild type *MMF1* sequence and the third line indicates the mutations created to change codon optimality but not the encoded amino acid. (**D**) Expression of the *MMF1* reporter with the wild type sequence (‘Original codon’) or the codon-optimized sequence (‘Optimized codon’) around the pause site in cells growing exponentially was evaluated by western blotting with antibodies to Flag or with antibodies to Egd2 to control for protein loading.

Most of the results presented so far were obtained with a reporter that expresses Mmf1 from an episome above the background of the endogenous *MMF1* gene. We used our published Ribo-Seq data that provides information about endogenous *MMF1* and *COX4* regulation in wild type cells and in cells lacking Not4 (36). From these experiments, we note that both *MMF1* and *COX4* mRNAs are up-regulated in the absence of Not4 (Figure 4A and Supplementary Figure S3A). We additionally observe that *MMF1* mRNA is translated with important ribosome pausing at codons 93 and 94, whilst no such pausing is detectable for *COX4*. Moreover, the pausing on *MMF1* mRNA is less effective in the absence of Not4, since ribosome footprints increase more after the pause site than before the pause site, in *not4Δ* (Figure 4B). *MMF1* pause site codons 93 and 94 are amongst the 15 most optimal codons in budding yeast, whereas the following codon 95 (that will be in the ribosomal A site for ribosomes pausing with codon 94 in the P-site) is one of the 15 least optimal codons (56). Codon 92 preceding the pause site is neither particularly optimal nor non-optimal. We replaced codons 92 and 95 with optimal codons encoding the same amino acids in the *MMF1* reporter (Figure 4C). This codon change was sufficient to lead to overexpression of the Mmf1 reporter (Figure 4D). Hence, not only ribosome pausing and Not4, but also codon optimality, is contributing to limit Mmf1 overexpression.

### Egd1 ubiquitination and Caf130 limit co-translationally *MMF1* expression

**Figure 5.**
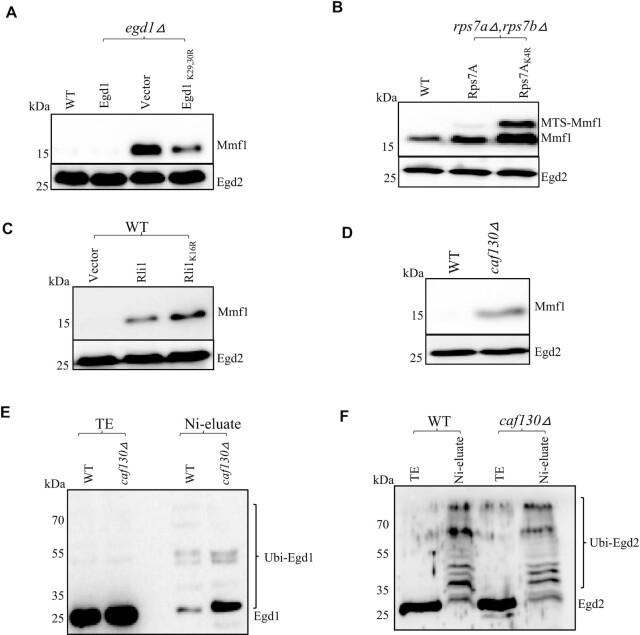
Ribosome-associated targets of Not4 ubiquitination, namely Egd1, Rps7A and Rli1, as well as Caf130, limit expression of the *MMF1* reporter. (**A**) Wild type (WT) or *egd1Δ* cells transformed with a plasmid expressing wild type HA-tagged Egd1 (Egd1), a control vector (vector) or a plasmid expressing an HA-tagged Egd1 derivative that does not get ubiquitinated (Egd1_K29,30R_) growing exponentially were tested for expression of the *MMF1* reporter. (**B**) Expression of the *MMF1* reporter was evaluated in WT cells and in cells expressing wild type (Rps7A) or non-ubiquitinated (Rps7A_K4R_) Rps7A from a plasmid to complement the deletion of genomic *RPS7A* and *RPS7B*. (**C**) Expression of the *MMF1* reporter in wild type cells transformed with a control plasmid or with a plasmid overexpressing Rli1 or a non-complementing Rli1 derivative with 16 lysine codons mutated to arginine was evaluated. (**D**) Wild type and *caf130Δ* cells growing exponentially were tested for expression of the *MMF1* reporter. In panels A–D the expression of the *MMF1* reporter was tested by western blotting with antibodies to Flag. Antibodies to Egd2 were used as loading control. (**E**, **F**) Wild type and *caf130Δ* cells expressing HA-tagged Egd1 from the endogenous *EGD1* locus and transformed with a plasmid 6His-tagged ubiquitin under the *CUP1* promoter were grown in the presence of 0.1 mM CuS0_4_. Ubiquitinated proteins were purified by nickel affinity chromatography and the presence of Egd1 in the total extract (TE) and nickel eluate (Ni-eluate) was tested with respectively antibodies to HA (E) or the presence of Egd2 with antibodies to Egd2 (F).

Both Egd1 and Not4 contribute to limit Mmf1 reporter overexpression. Egd1 is a substrate for the ubiquitin ligase activity of Not4 and ubiquitinated residues have been characterized (40,57). We thus tested expression of the Mmf1 reporter in wild type cells, or in *egd1Δ* cells transformed with either an empty vector, a vector expressing wild type Egd1, or a plasmid expressing the non-ubiquitinated Egd1_K29,30,R_ derivative (57). The Mmf1 precursor was overexpressed in *egd1Δ* as expected, and this was complemented by wild type Egd1, but not by the non-ubiquitinated Egd1 (Figure 5A).

We recently determined that Not4 ubiquitination of Rps7A and overexpression of another target of Not4 ubiquitination, Rli1, wild type or with 16 mutated lysine codons, increased translation of a reporter with a stalling sequence (36). Because ribosome pausing appears relevant for the response that limits the overexpression of the *MMF1* reporter, we tested the impact of non-ubiquitinated Rps7A on expression of the *MMF1* reporter. Mmf1 was increased in the non-ubiquitinated Rps7A mutant (Figure 5B). Similarly, Rli1 overexpression increased the over-expression of the *MMF1* reporter (Figure 5C) but it had no effect on Cox4 expression (Supplementary Figure S3B).

We have observed using Not5 affinity purification that Egd1 co-purifies with the Ccr4–Not complex from wild type cells, but it does not co-purify with it from cells lacking Caf130, another subunit of the Ccr4–Not complex, as previously reported by others (58) and confirmed recently (59). The Mmf1 reporter was also overexpressed in cells lacking Caf130 (Figure 5D), whilst Cox4 was unaffected (Supplementary Figure S3C). Since Egd1 ubiquitination by Not4 is important to repress Mmf1 overexpression, and Caf130 is important for co-purification of Egd1 with the Ccr4–Not complex, we determined whether ubiquitination of Egd1 was impaired in cells lacking Caf130. We transformed a plasmid expressing His-tagged ubiquitin from the *CUP1* promoter in *caf130Δ* cells expressing HA-tagged Egd1. After induction with copper, we affinity purified ubiquitinated proteins on a nickel resin. Total proteins and affinity-purified proteins were analyzed by western blotting for Egd1 with antibodies to HA (Figure 5E). Egd1 ubiquitination was not abolished in cells lacking Caf130. However, in cells lacking Caf130 there was higher accumulation of lower molecular weight ubiquitinated forms and reduced accumulation of higher molecular weight ubiquitinated forms of Egd1, suggesting reduced turnover of ubiquitinated Egd1 in *caf130Δ*. The ubiquitination of Egd2, the heterodimeric partner of Egd1 in the NAC complex, was not detectably affected (Figure 5F). Notably, Mmf1 was overexpressed to similar levels whether only Egd1 was deleted, versus if all NAC subunits (Egd1, the other β NAC subunit Btt1, and Egd2) were deleted (Supplementary Figure S4A). Sam37 has been proposed to cooperate with NAC to mediate early stages of mitochondrial protein import (13) and interestingly, the *MMF1* but not *COX4* reporter protein and mRNA, were overexpressed in cells lacking Sam37 (Supplementary Figure S4B and C).

### RQC, as well as Cis1, Hsp104 and autophagy limit overexpression of Mmf1

**Figure 6.**
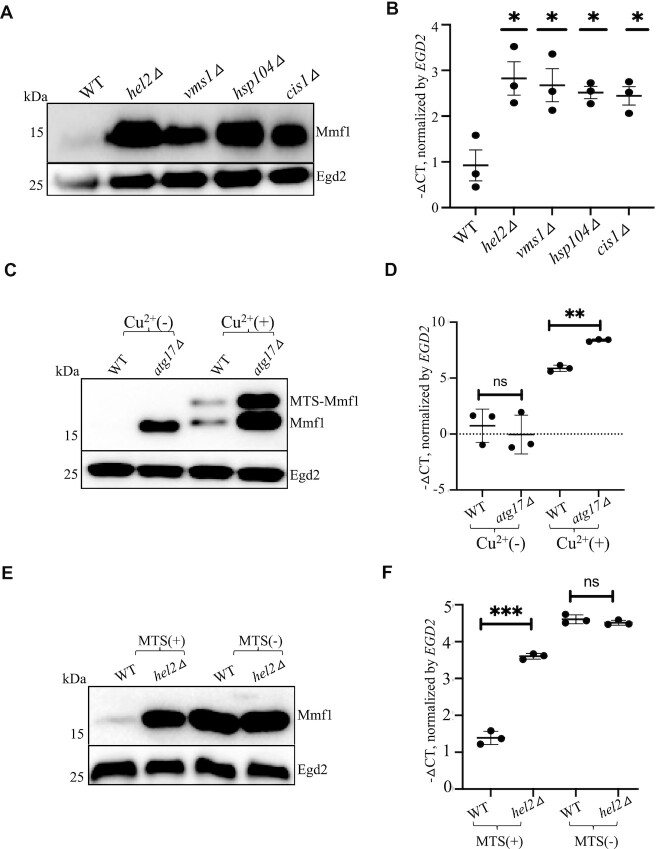
An integrated quality control response regulates expression of the *MMF1* reporter mRNA and protein. (**A**, **B**) Expression of the *MMF1* protein and mRNA was tested in wild type cells (WT) and in cells lacking *HEL2*, *VMS1*, *HSP104* or *CIS1* growing exponentially by western blotting with antibodies to Flag or with antibodies to Egd2 used as loading control (A) or by RT-qPCR (B). For the mRNA, the levels were normalized to *EGD2* and the results are expressed as – ΔCT values in the different strains relative to WT. The level of significant change, relative to WT is indicated with asterisks using a two-sided, Welch, unpaired *t*-test (*n* = 3). (**C**, **D**) Expression of the *MMF1* reporter was tested in WT and *atg17Δ* cells growing exponentially, before and after a 10 min copper induction for protein (C) and mRNA (D) levels. (**E**, **F**) Expression of the *MMF1* reporter with or without MTS was tested in WT or in cells lacking *HEL2* growing exponentially as in panels A and B, respectively.

As mentioned above, ribosome pausing appears relevant for limitation of *MMF1* overexpression. Long lasting ribosome pausing can cause ribosome collisions and induce the RQC response. We thus tested expression of the reporters in wild type cells and in cells lacking Hel2, a major effector of RQC, or lacking Vms1, the tRNA hydrolase that antagonizes Rqc2. Mmf1 was overexpressed in both mutants (Figure 6A). RQC is accompanied by NGD that involves both 5’ to 3’ and 3’ to 5’ mRNA degradation, for which Xrn1 and Ski2 contribute. *MMF1* was overexpressed in cells lacking either protein (Supplementary Figure S5A). *COX4* was not affected by the absence of *XRN1* but interestingly it was a little up-regulated in the absence of *SKI2*.

As mentioned above, many QC pathways exist to avoid accumulation of proteins that arrive at the mitochondria, either overexpressed precursor proteins, mistargeted proteins or misfolded and defective proteins. We tested the role played by components of these QC responses, starting with Cis1. Cis1 associates with the mitochondrial translocase and is known to be key to reduce the accumulation of mitochondrial precursor proteins with the Cis1-interacting AAA^+^ adenosine triphosphatase that contributes to extract proteins from the outer membrane (MitoCPR) (22). Mmf1 was overexpressed in cells lacking Cis1 (Figure 6A), but not in cells lacking Msp1 (Supplementary Figure S5B). Thus, the regulation of Mmf1 overexpression involves Cis1 but by a mechanism distinct to ‘MitoCPR’. Mmf1 was also overexpressed in cells lacking the Hsp104 disaggregase (Figure 6A). In all these cases, not only Mmf1 protein, but also *MMF1* mRNA, was overexpressed (Figure 6B). Cox4 expression was not affected in any of these mutants (Supplementary Figure S5C–E).

We also tested whether mitophagy that removes aged and damaged mitochondria (26) contributed to limit Mmf1 overexpression using a strain lacking Atg32, the receptor for mitophagy. However, Mmf1 levels were unaltered in *atg32Δ* (Supplementary Figure S5F). Mitophagy is a selective type of autophagy, so we tested whether autophagy contributed to limit Mmf1 overexpression, using cells lacking the Atg17 scaffold protein. Mmf1 was indeed overexpressed in cells lacking Atg17, both the protein (Figure 6C) and the mRNA (Figure 6D), though in this latter case it was only clearly visible after copper induction. Cox4 was unaffected in cells lacking Atg17 (Supplementary Figure S6A). Many other autophagy mutants were tested, with the same effect on Mmf1 but not Cox4 overexpression (Supplementary Figure S6B-C).

Expression of *MMF1* without its MTS was not increased in any of the mutants of these different QC pathways (Supplementary Figure S6D). Even in the case of cells lacking Hel2, expression of *MMF1* without its MTS was not increased, either at the protein (Figure 6E) and or at the mRNA (Figure 6F) level.

These results indicate that many QC pathways work together to limit *MMF1* mRNA, and hence synthesis and accumulation of the Mmf1 precursor, as long as the Mmf1 has its mitochondrial targeting sequence.

### The Mmf1 MTS contributes to localize the *MMF1* mRNA to mitochondria

**Figure 7.**
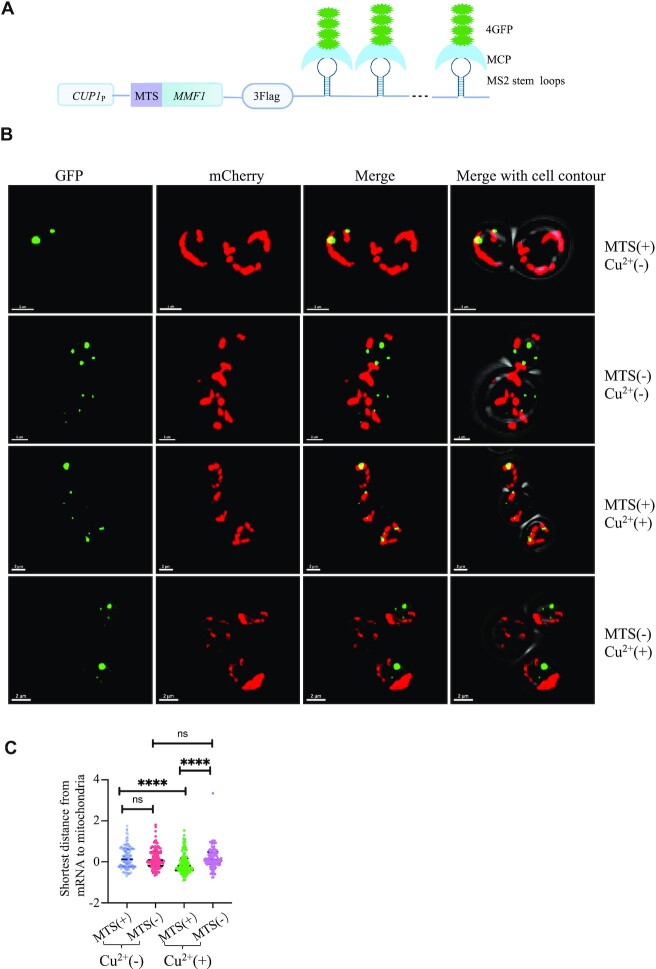
The Mmf1 MTS contributes to the localization of the *MMF1* mRNA to the mitochondria. (**A**) Cartoon of the *MMF1* reporter with inserted MS2 stem loops in the 3’UTR that can be recognized by MS2 binding protein (MCP) fused to 4 GFP. (**B**) Wild type cells with an integrated su9-mCherry reporter to follow mitochondria were transformed with the plasmid expressing the *MMF1* reporter with or without its MTS as indicated. Cells were grown to exponential phase, and induced (+) or not (–) with copper (Cu^2+^) for 10 min, then were fixed and visualized at the confocal microscope to see GFP (left panels), Su9-mCherry (second to left panels), and the merged signal (second to right panels), with the contour of the cells indicated (far right panels). Representative images of 2–3 cells are shown. (**C**) To determine the distance between mRNAs and mitochondria and evaluate mRNA localization at the mitochondrion, the Imaris software (version 9) was used, with its sport model generating the 3D model of mRNA and its surface model building the 3D model of mitochondria. Statistical analysis was performed using Prism9, with a two-tailed unpaired t-test, with Welch's correction. Each sample was assessed for more than 100 spots.

All of the results above raise the question of how Not4 and the Mmf1 MTS together with the co-translational import machinery repress overexpression of the *MMF1* reporter. Our observations are consistent with the possibility that the Mmf1 MTS together with Not4 might contribute to target the *MMF1* mRNA to the co-translational import machinery. To determine anchoring of the *MMF1* mRNA to the co-translational import machinery, we inserted new generation MS2 stem loops (sl) (60) into the 3’UTR of the *ADH1* terminator on the reporter carrying the *MMF1* ORF, with or without its MTS (Figure 7A). We transformed this reporter into cells expressing the MS2-stem loop binding protein (MCP) fused to 4 GFPs and expressing the matrix marker Su9-mCherry (61). The *MMF1* reporter mRNA was detectable with the bound MCP-4GFP fluorescent protein with (Figure 7B, upper left panel) and without MTS (second from the top left panel), and as expected was present at higher levels in the latter case. The mitochondria were clearly detectable via the Su9-mCherry fluorescence (second to left panels). Merging of the signals allowed us to evaluate the extent of co-localization. The experiment was performed before (upper 2 lines) and after (lower 2 lines) copper induction. Interestingly copper induction increased the amount of mRNA detectable for the *MMF1* reporter with MTS but not without MTS (when it is already overexpressed). Before copper induction, some co-localization was detectable for the *MMF1* with its MTS, but it did not appear statistically significantly different from the *MMF1* without its MTS. However, the level of these two different reporter mRNAs was very different before copper induction making it difficult to conclude. After copper induction, the co-localization of the *MMF1* mRNA with its MTS and mitochondria was significantly higher compared to that of the *MMF1* mRNA without its MTS (Figure 7C).

## DISCUSSION

### Targeting and pausing for quality control at the mitochondria outer membrane

**Figure 8.**
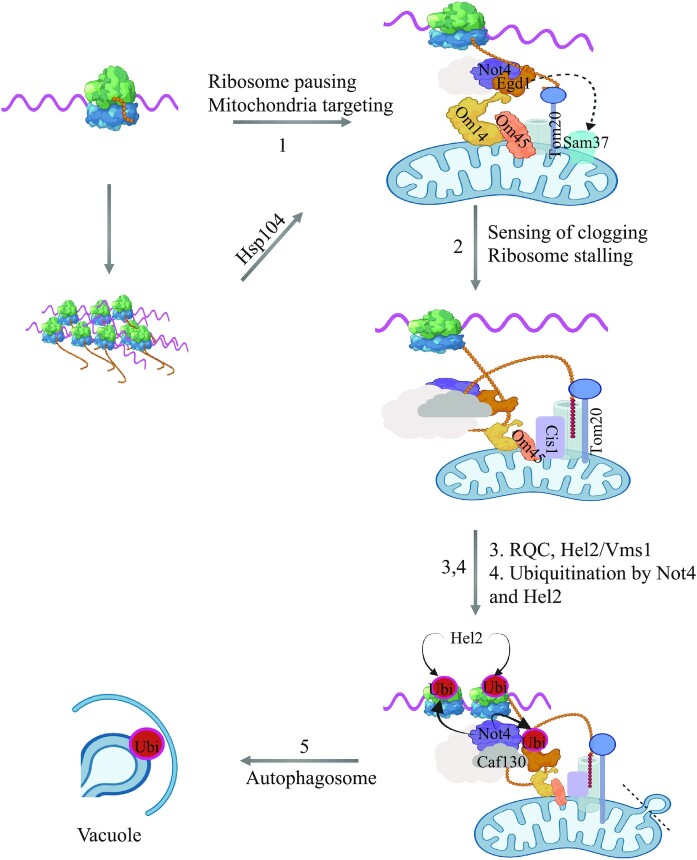
Model for limitation of translationally arrested mRNAs at the mitochondrial surface: Mito-ENCay. Overexpressed *MMF1* mRNA is targeted to the mitochondria via its nascent chain where its translation undergoes pausing, and both induction of the RQC/NGD and autophagy pathways reduce mRNA levels to limit protein synthesis and accumulation of Mmf1. This system relies on ribosome pausing and the co-translational targeting of the *MMF1* mRNA to the mitochondria via the Mmf1 nascent chain, the Egd1 chaperone, Om14, (alternatively Sam37), Om45 and Tom20 at the mitochondrial OM and Not4 of the Ccr4–Not complex (step 1). The Hsp104 disaggregase also plays a role, likely for targeting, if the nascent chain starts aggregating before targeting is ensured. Then, at the mitochondrial OM, ribosome pausing is increased if the import of the nascent chain is slowed down. Sensing is likely to be contributed to by Cis1, Om45 and Tom20 (step 2). Upon increased ribosome pausing, ribosome collisions will induce RQC and NGD initiated by ubiquitination by Hel2 (step 3), and result in degradation of the *MMF1* mRNA to limit new Mmf1 synthesis and accumulation. The limitation of *MMF1* mRNA is additionally provided by further ubiquitination of RNC-associated proteins by both Hel2 and Not4 (step 4), then autophagy, whereby vesicles of mitochondria fragments, rich in OM with docked RNCs and accumulated ubiquitinated proteins, are targets for autophagosome formation and targeting to the vacuole for degradation (step 5).

In this work we show that budding yeast cells growing in glucose with limited need for mitochondria can mobilize an integrated QC response to avoid overexpression of the Mmf1 mitochondrial precursor induced from an episome. We have called this mechanism Mito-ENCay (Figure 8). According to our model, Mito-ENCay relies on the co-translational targeting of the *MMF1* mRNA to the MOM via the MTS of the Mmf1 nascent chain and its NAC-bound chaperone docking the RNC onto the MOM via its receptor Om14 or Sam37 (step 1). In addition, the Hsp104 disaggregase is important for Mito-ENCay, maybe by disaggregating nascent chains during translation to allow more efficient targeting of the *MMF1* RNC to the MOM (Figure 8, step 1).

Previous studies analyzing translation of nuclear-encoded mitochondrial mRNAs expressed at physiological levels from their endogenous loci in glucose did not detect *MMF1* as an mRNAs being translated at the MOM (5,62). The difference is that in this work we look at how the cell copes with additional *MMF1* mRNA expressed from an episome. It could be that co-translational MOM targeting occurs only when *MMF1* mRNA is in excess, or that it is only detected under these conditions. In this regard, our imaging of the *MMF1* mRNA showed that its detectable presence at the MOM was significantly increased when *MMF1* mRNA expression was increased (Figure 7). One explanation for this can be that under conditions of *MMF1* overexpression the presence of the RNC at the MOM is longer lasting. Indeed, we see that *MMF1* is translated with ribosome pausing. This pausing may enable co-translational targeting of the mRNA to the MOM or instead be the consequence of mRNA targeting to the MOM. Regardless, under low levels of expression, the nascent Mmf1 chain is likely to enter the import channel rapidly, resulting in a lift of ribosome pausing and the mRNA is rapidly released from the MOM at the end of translation. Instead, at high levels of the *MMF1* mRNA, one can imagine that the import channel becomes overwhelmed by the Mmf1 precursor and that ribosome pausing for *MMF1* RNCs arriving at the MOM will be sustained. Thereby, the mRNA has more chances to be detected at the MOM and ribosome collisions can occur resulting in RQC. It is interesting to note that accumulation of ribosome footprints 30 nucleotides and 60 nucleotides upstream of the major ribosome pause site on *MMF1* is already detectable under low levels of *MMF1* expression in glucose (Figure 4A). This suggests that ribosome collisions may already occur for *MMF1* expressed from its endogenous locus in glucose.

The *MMF1* MTS plays a key role in Mito-ENCay. Indeed, *MMF1* mRNA without MTS is not regulated by Mito-ENCay and it is overexpressed. The MTS could be essential for mitochondrial targeting, or the MTS might be important for ribosome pausing, in turn essential for targeting to the MOM. These two possibilities may not be independent. Indeed, ribosome pausing may occur because of the docking of the RNC onto the MOM, or ribosome pausing might give more chance to the RNC to be targeted to the MOM by slowing down translation. It has already been demonstrated that slowing down translation gives more chance for co-translational targeting to the MOM (5), and here we show that codon optimality around the ribosome pause site is important for Mito-ENCay, supporting the idea that translation elongation dynamics is an important factor. Deletion of the RQC machinery does not increase the already high levels of *MMF1* mRNA without an MTS. This indicates that there is no ribosome stalling or ribosome collisions for an *MMF1* mRNA without its MTS. This could be because the MTS directly affects ribosome pausing or because ribosome stalling occurs at the MOM. We favor this latter hypothesis. First, the docking site on the MOM for the chaperone that binds the nascent chain is important for Mito-ENCay supporting a role for targeting to the MOM in MitoENCay. Second, the MTS is only a few codons, and the major ribosome pause site on *MMF1* is nearly 90 codons after the MTS (Figure 4A). Finally, the MTS can work when fused to the *COX4* coding sequences instead of the Cox4 MTS. A model whereby this is because the MTS contributes to dock the RNC onto the MOM seems the most likely.

We determine that the Not4 subunit of the Ccr4–Not complex is important for Mito-ENCay in a manner epistatic to the MTS. Not4 is important for effective ribosome pausing when *MMF1* mRNA is produced from the endogenous locus in glucose and ubiquitination of its ribosome-associated targets, namely Rli1 and Rps7A that regulate ribosome pausing (36), contribute to Mito-ENCay. Like the MTS, Not4 might play a direct role in targeting, for instance via its interaction with NAC that binds the nascent chain, or it could play a role in translation elongation dynamics, enabling co-translational targeting of the *MMF1* RNC to the MOM. The fact that *MMF1* solubility is increased in the absence of Not4 is compatible with both models. However, we favor the latter one. Indeed, Not4-associated ribosomes are post-translocation ribosomes with an empty A site (37) more likely to occur when the codon in the A site is non-optimal, and the first codon after the *MMF1* ribosome pause site is a non-optimal codon that is important for Mito-ENCay. Furthermore, we have proposed that the binding of Not proteins to the translating ribosome may result in tethering of the RNC to condensates, in which translation elongation dynamics is different (36).

Another subunit of the Ccr4–Not complex, Caf130, is important for Mito-ENCay. Not much is known about the function of Caf130, but it is necessary for stable association of NAC with the complex ((58,59) and our own unpublished results). Hence it might contribute to recruit the Ccr4–Not complex to the NAC-associated nascent chain, or *vice-versa* recruit NAC to the Not4-associated RNC, or finally it may stabilize the complex of the RNC with NAC and Not4 (Figure 8, step 1). Caf130 might also contribute to subsequent steps of Mito-ENCay enabling effective ubiquitination of NAC (Figure 8, step 4).

Degradation of excess *MMF1* mRNA by Mito-ENCay implies not only that there is targeting of the *MMF1* mRNA to mitochondria during translation (Figure 8, step 1), but also that there is a mechanism that regulates ribosome pausing at the MOM (Figure 8, step 2). This raises the question of what determines the lift of ribosome pausing versus a stalling of translation. We observe that Cis1, already described to be important for detection of excess and/or aberrant precursor proteins at the MOM for ‘Mito-CPR’ (22), is needed for Mito-ENCay. For Mito-CPR it works together with the Msp1 ATPase to extract proteins from the MOM for degradation in the cytoplasm by the proteasome. Instead, for Mito-ENCay Msp1 is not relevant. Moreover, while we detect that some of the overexpressed Mmf1 and Cox4 is degraded in the cytoplasm by the proteasome, mutants of the proteasome have no impact on the level of the *MMF1* mRNA, unlike the *cis1Δ* mutant. Instead, Om45 that interacts with Om14, Sam37 and Tom20 responsible for recognition and initial import steps for all mitochondrially directed proteins, all contribute to Mito-ENCay (Figure 8, step 2). These factors are all candidates, with Cis1, for a role in regulation of ribosome pausing at the MOM according to the availability of the import channel.

If ribosome pausing at the MOM is sustained, then the risk for ribosome collisions increases and the RQC response will be induced. Consistently, the RQC response factors Hel2 and Vms1 are important for Mito-ENCay (Figure 8, step 3). It seems likely that the RQC can be rapidly overwhelmed and this can result in the induction of an additional QC response, namely autophagy (Figure 8, steps 4 and 5). Increased ubiquitination of RNC-associated proteins, probably by both Hel2 and Not4, appears to be a major contributor to this additional QC. Hel2 ubiquitinates ribosomal proteins in response to collided ribosomes (38,63), including Rps7A first mono-ubiquitinated by Not4, and it could be that Hel2 can similarly polyubiquitinate Egd1 and Rli1 after Not4 mono-ubiquitination in specific QC conditions. Protein ubiquitination is necessary in many types of selective autophagy as a mark for cargo recognition and a signal for process initiation by recruitment of specific autophagy adaptor proteins (reviewed in (64)). In addition, in a recent study a role for Not4 ubiquitination of Rli1 in the context of paused RNCs at the MOM for mitophagy in flies has been proposed (65). Ultimately fission and degradation of mitochondrial vesicles with highly ubiquitinated RNCs on their membrane will be degraded by autophagy (Figure 8, step 5), thereby preserving regions of the mitochondria without stalled RNCs. In this context it is interesting to note that Not4-dependent ubiquitination of Rps7A is important for *HAC1* translational up-regulation in response to ER stress, and the presence of the *HAC1* mRNA at the ER is necessary for this up-regulation (66). *HAC1* mRNA solubility, like the solubility of *MMF1*, increases upon Not4 depletion (39). Hence, it could be that Not4 contributes to ER targeting of the *HAC1* mRNA.

An important question is whether all mitochondrial mRNAs can be targets for Mito-ENCay when overexpressed, or whether this mechanism is specific for some mRNAs. Our results show that the *COX4* ORF is not a target for Mito-ENCay, but it can be repressed if the *MMF1* MTS replaces the *COX4* MTS. This suggests that maybe only mRNAs with specific N-terminal MTS sequences can be subject to Mito-ENCay. We noted that overexpressed Cox4 turns over very rapidly, and from this we can imagine that its overproduction does not endanger cellular proteostasis. So it could be that Mito-ENCay is a mechanism that has evolved to limit overexpression of mRNAs encoding proteins that are likely to aggregate and/or block the import channel. Intriguingly, solubility of *COX4* mRNA is also regulated by Not4, and overall ribosome footprints are increased on *COX4* mRNA in *not4Δ*. Moreover, *COX4* mRNA levels are also increased in *not4Δ*. It could be that solubility of *COX4* mRNA is regulated by Not condensates (35) that might also play a role in production of Cox4, for instance for effective interaction of nascent Cox4 with cytosolic chaperones or post-translational targeting of Cox4 to mitochondria. Furthermore, *COX4* regulation by the Not proteins might also depend upon 5’ or 3’UTR sequences rather than on the coding sequence as was tested in this study.

## DATA AVAILABILITY

No new data was generated in this work. The data analyzed was already deposited in public data bases.

## Supplementary Material

gkad299_Supplemental_File
