# Supplementary material for: Not4-dependent targeting of MMF1 mRNA to mitochondria limits its expression via ribosome pausing, Egd1 ubiquitination, Caf130, no-go-decay and autophagy
Source: Nucleic Acids Res. 2023 Apr 24;51(10):5022–39. doi: 10.1093/nar/gkad299 (PMC10250226; doi:10.1093/nar/gkad299)
Supplement: gkad299_Supplemental_File [file gkad299_supplemental_file.pdf]

S1

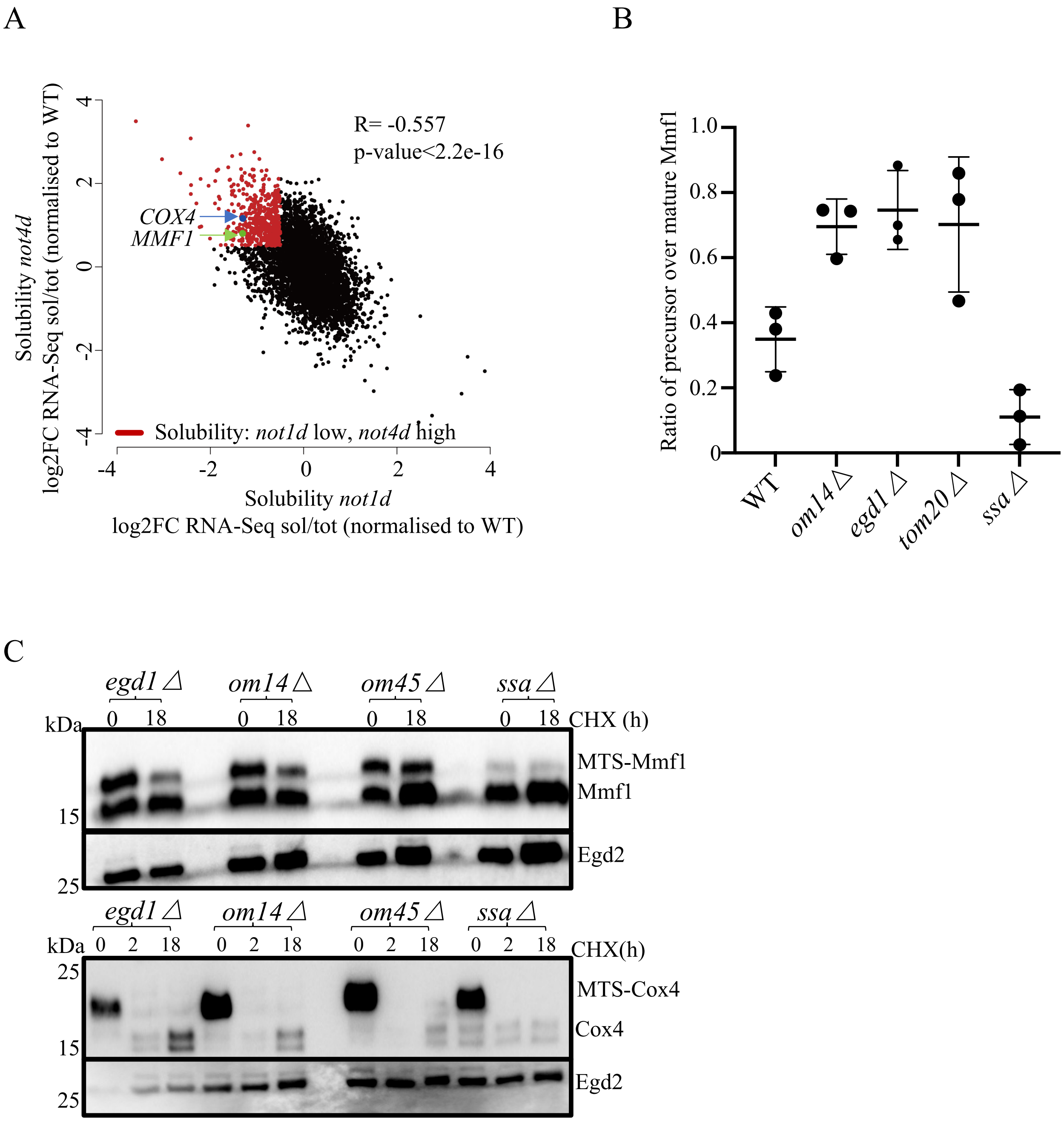

Figure S1. A. Indication of *MMF1* and *COX4* mRNAs on a scatterplot comparing changes in mRNA solubility before and after Not1 and Not4 depletion (43). mRNAs more soluble upon Not4 depletion but less upon Not1 depletion are indicated in red. B. Quantification of Mmf1 precursor relative to mature Mmf1 after copper induction for experiment in Figure 1D. C. Expression of the Mmf1 and Cox4 reporters in the indicated strains growing exponentially after a 10 min copper induction (0) followed by treatment with CHX for 2 or 18h as indicated, by western blotting with antibodies to Flag or Egd2 as loading control.

S2

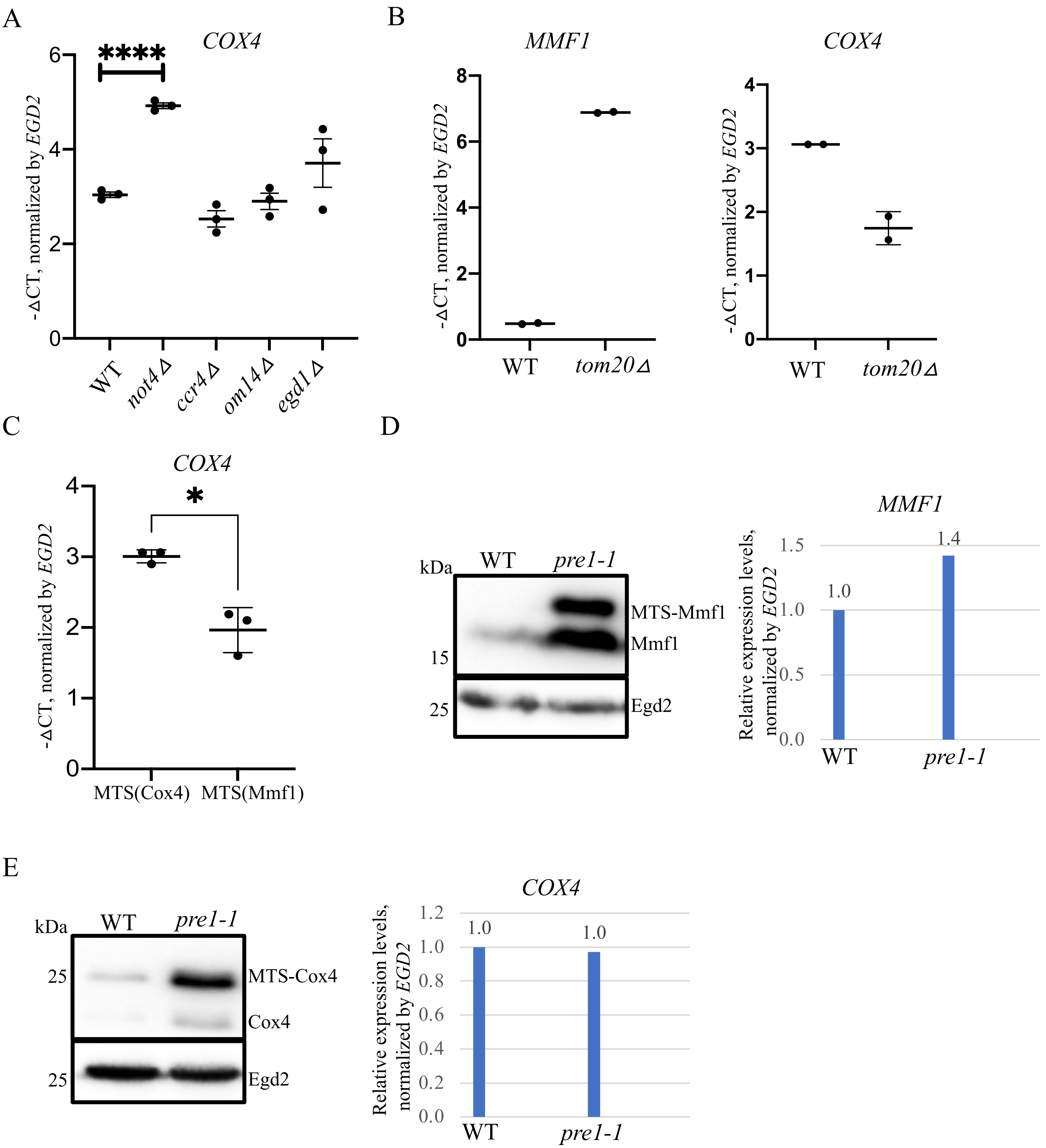

Figure S2. **A**. Analysis of *COX4* reporter mRNA levels by RT-qPCR in the indicated strains. The *EGD2* mRNA was used as a control for loading. The *COX4* reporter mRNA was plotted to show means +/- SD of – ΔCT values. The level of significant change, relative to WT is indicated with asterisks using a two-sided, Welch, unpaired t-test (n = 3). (**B**) Analysis of *MMF1* and *COX4* reporter mRNA in wild type and *tom20Δ* cells as indicated growing exponentially by RT-qPCR. Results are of biological duplicate experiments. (**C**) Analysis of *COX4* reporter mRNA levels corresponding to the experiment in **Figure 2D** by RT-qPCR as in panel A. (**D**) Analysis of the *MMF1* reporter in wild type cells or the proteasome *pre1-1* mutant growing exponentially, by western blotting with antibodies to Flag or to Egd2 for loading control (left panel) or by RT-qPCR as in panel B (right panel). **(E)** Analysis of the *COX4* reporter as for *MMF1* in panel **D**.

S3

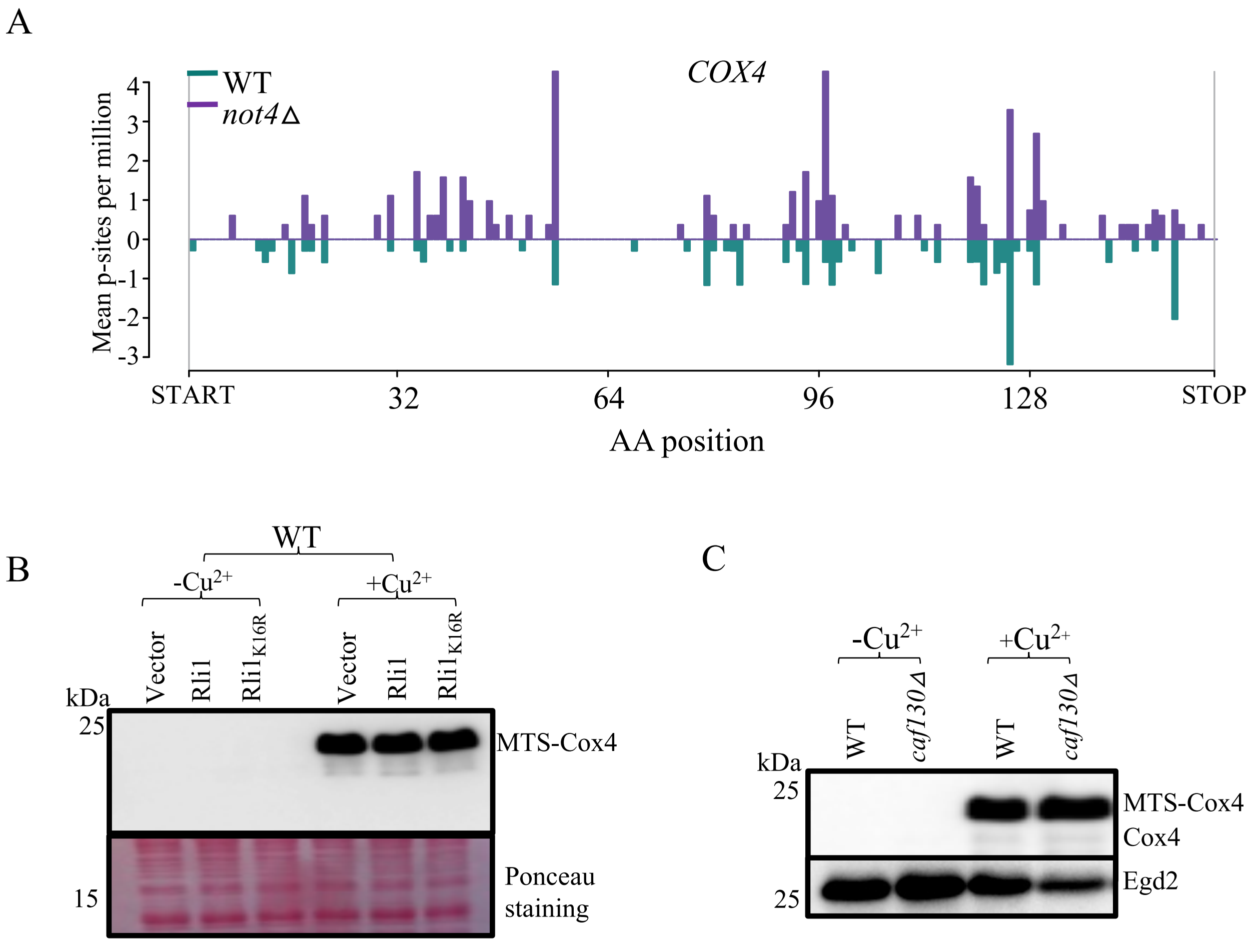

Figure S3. A. Profiles of ribosome footprints (P-site depth plots) on *COX4* with footprints in wild type cells in green and those in *not4*△ in purple. The number of P-sites, per million genome-wide for each sample, covering each CDS codon with corresponding amino acid position indicated (AA position) is calculated, averaged for each condition and plotted. **B**. Expression of the Cox4 reporter in wild type cells growing exponentially with or without overexpression of wild type or K16R-mutated Rli1, before and after copper induction, by western blotting with antibodies to Flag. The ponceau staining is shown as loading control. **C**. Expression of the Cox4 reporter in wild type cells or cells lacking Caf130 growing exponentially before and after copper induction is analyzed by western blotting with antibodies to Flag or to Egd2 for loading control.

S4

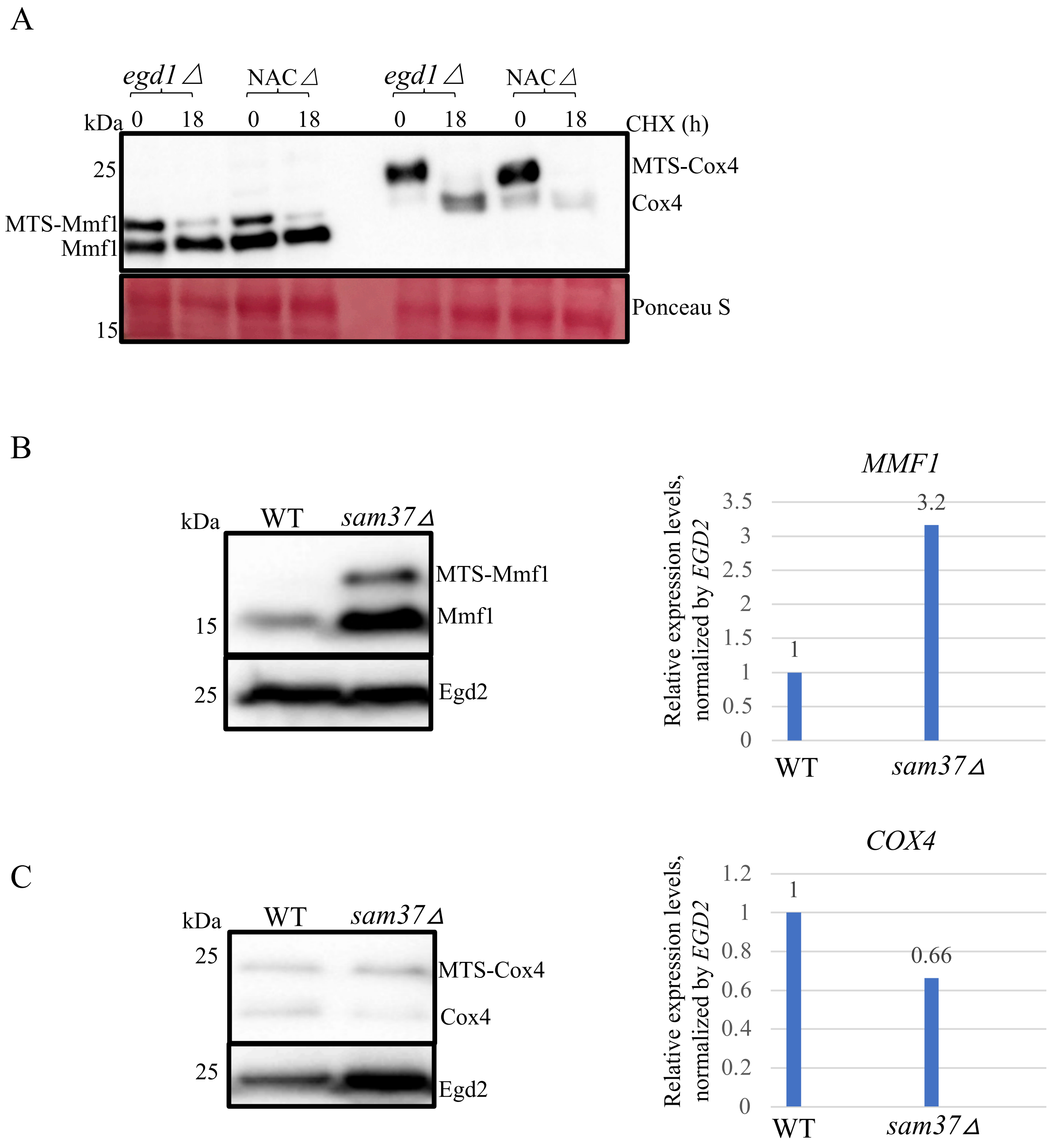

Figure S4. **A**. Expression of the Mmf1 and Cox4 reporters is compared in cells lacking Egd1 or all 3 NAC subunits and growing exponentially after a 10 min copper induction treated or not with CHX for 18 h, by western blotting with antibodies to Flag or to Egd2 for loading control. **B**. Expression of the *MMF1* reporter is compared in wild type cells and in cells lacking Sam37 for protein levels by western blotting with antibodies to Flag or to Egd2 for loading control (left) or for mRNA by RT-qPCR (right). Results are for a single experiment. **C**. The same experiment as in panel B was done to evaluate expression of the *COX4* reporter.

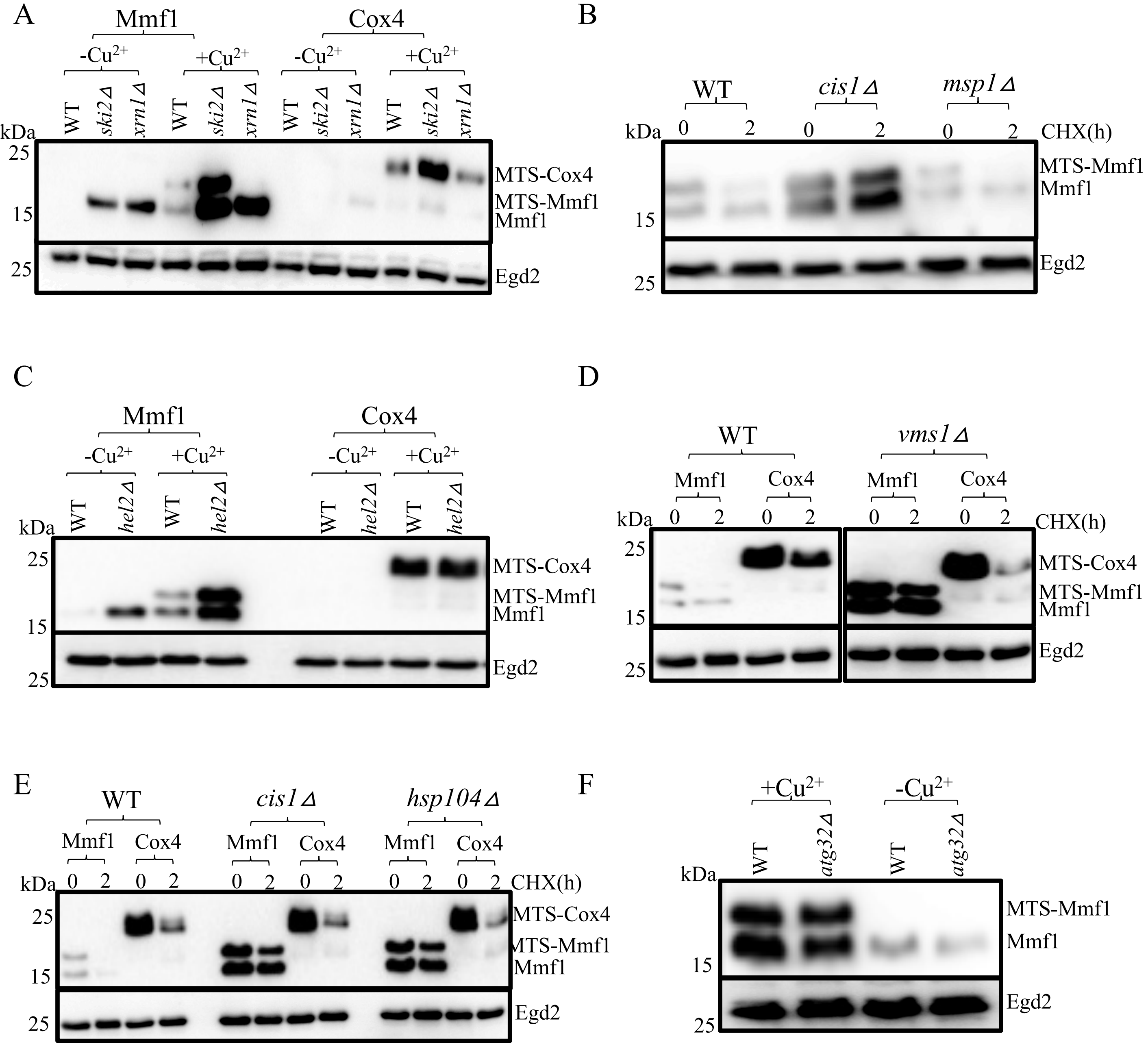

Figure S5. **A**. Expression of the Mmf1 and Cox4 reporters were tested in wild type cells, or in cells lacking Ski2 or Xrn1 before and after copper induction by western blotting with antibodies to Flag or to Egd2 for loading control. **B**. Expression of the Mmf1 reporter was compared in wild type cells, or in the *cis1Δ* or *msp1Δ* mutants after 10 min copper induction or 2 hours after adding CHX, by western blotting with antibodies to Flag or to Egd2 for loading control. **C**. Expression of the Mmf1 or Cox4 reporters were tested in wild type or *hel2Δ* cells before and after copper induction by western blotting with antibodies to Flag or to Egd2 for loading control. **D**. Expression of the Mmf1 or Cox4 reporters were tested in wild type or *vms1Δ* cells after a 10 min copper induction or 2 hours after adding CHX by western blotting with antibodies to Flag or to Egd2 for loading control. **E**. Expression of the Mmf1 and Cox4 reporters were tested in wild type cells, or in *cis1Δ* and *hsp104Δ* cells after a 10 min copper induction and 2 hours after adding CHX, by western blotting with antibodies to Flag or to Egd2 for loading control. **F.** Expression of the Mmf1 reporter was tested before and after copper induction in wild type cells or cells lacking Atg32 by western blotting with antibodies to Flag or to Egd2 for loading control.

S6

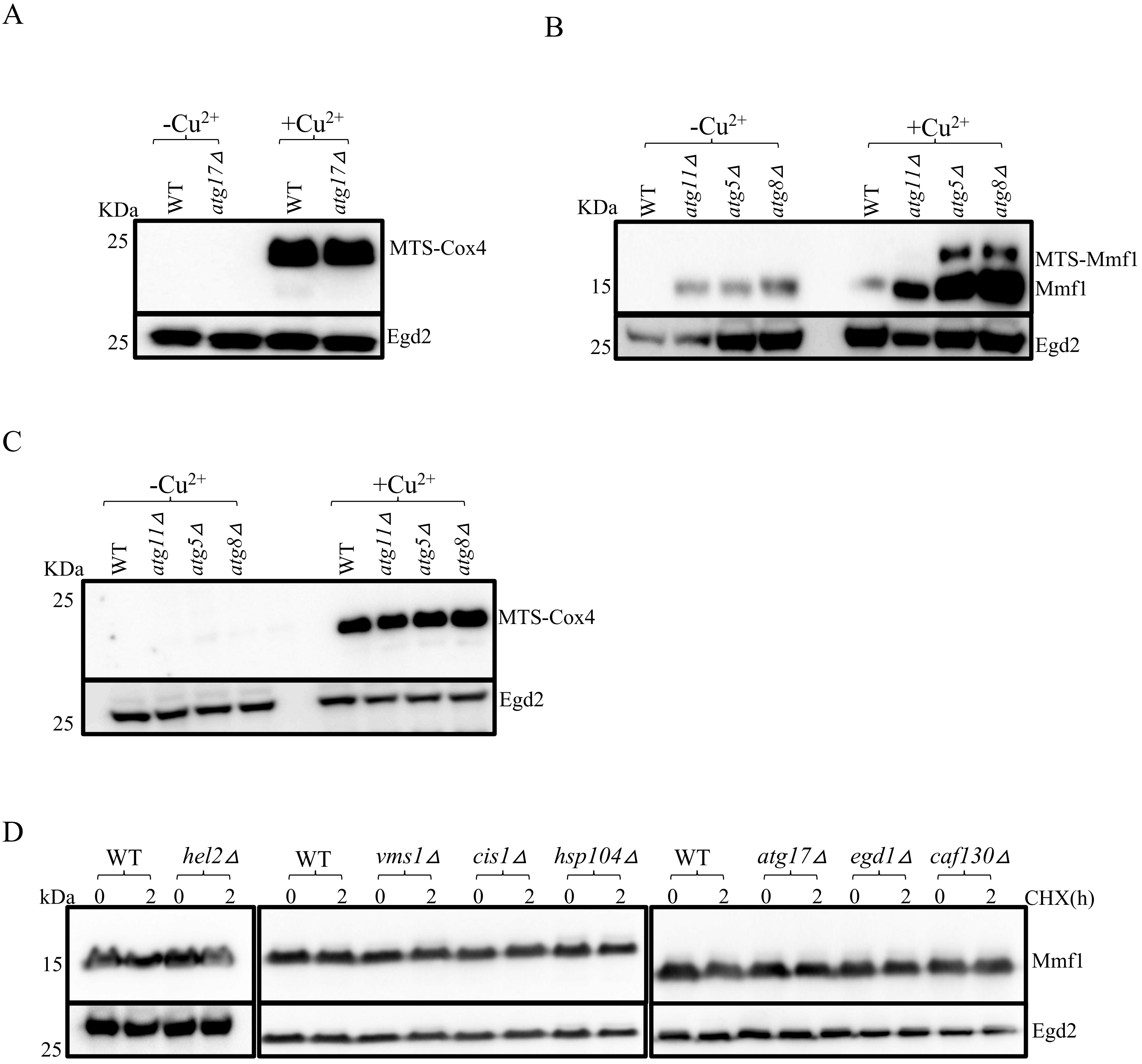

Figure S6. **A**. Expression of the Cox4 reporter was tested before and after copper induction in wild type cells or cells lacking Atg17 by western blotting with antibodies to Flag or to Egd2 for loading control. **B**. Expression of the Mmf1 reporter was tested in the indicated strains before and after copper induction by western blotting with antibodies to Flag or to Egd2 for loading control. **C**. Expression of the Cox4 reporter was tested as in panel B. **D**. Expression of the Mmf1 reporter without the MTS was tested in the indicated strains growing exponentially by western blotting with antibodies to Flag or to Egd2 for loading control.

S7

1B

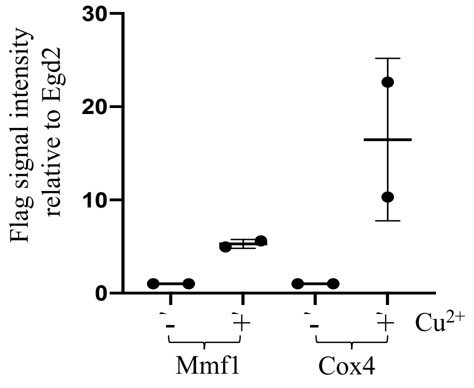

2C

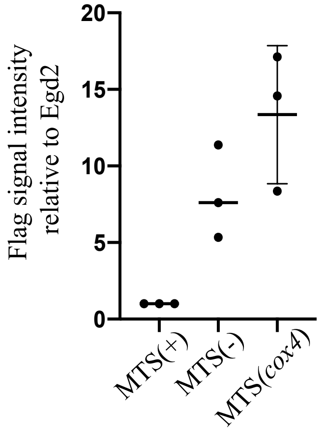

2D

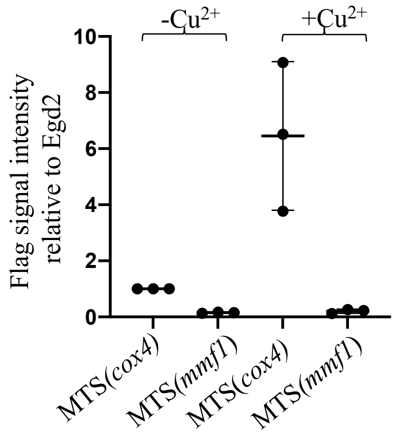

3A

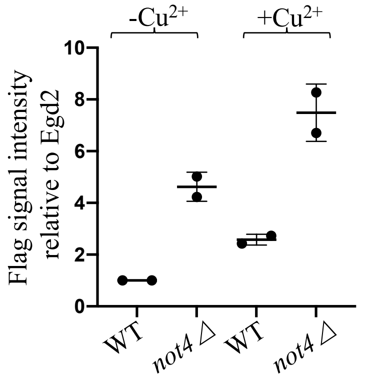

4D

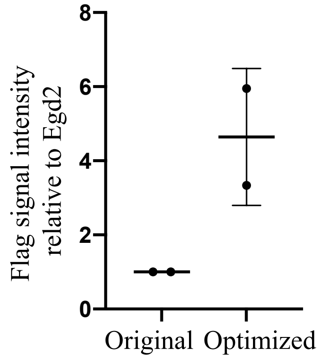

5D

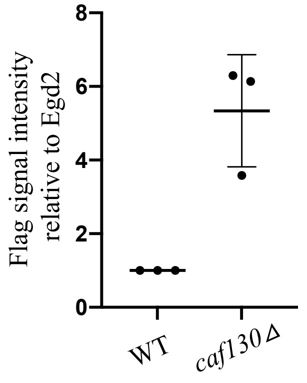

6C

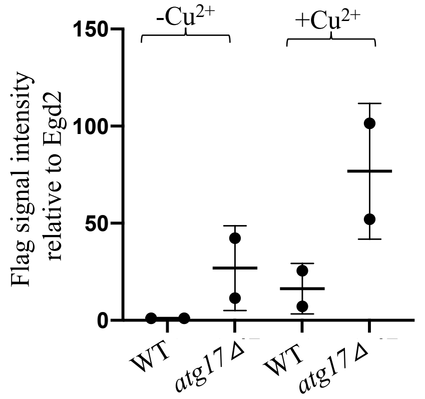

Figure S7. The relevant western blots shown in the main figures were performed at least in biological triplicates and for some of these quantification as indicated is shown (Figure 1B, 2C, 2D, 3A, 4D, 5D, 6C).

Internal number	Plasmids	Reference
pMAC594	ADH1p-EGD1-HA-TRP1	Panasenko et al., 2009
pMAC634	ADH1p-egd1-K29,30R-HA-TRP1	Panasenko et al., 2009
pMAC684	NOT4p-Myc6-NOT4-ADH1t	Panasenko and Collart, 2012
pMAC721	NOT4p-Myc6-not4-1-430-ADH1t	Panasenko and Collart, 2012
pMAC751	NOT4p-Myc6-not4-235-587-Q560R	Panasenko and Collart, 2012
pMAC1200	CUP1p-COX4-Flag-ADH1t-URA3	This work
pMAC1211	CUP1p-MMF1-Flag-ADH1t-URA3	This work
pMAC1321	CUP1p-Flag-rli1-K16R-LEU2	Pansenko et al., 2019
pMAC1322	CUP1p-Flag-RLI1-LEU2	Panasenko et al., 2019
pMAC1327	CUP1p-MTS-less-MMF1-Flag-ADH1t-URA3	This work
pMAC1328	CUP1p-Cox4MTS-MMF1-Flag-ADH1t-URA3	This work
pMAC1342	CUP1p-MMF1-Flag-ADH1t-ura3::LEU2	This work
pMAC1365	CUP1p-MMF1-Flag-MS2bps-URA3	This work
pMAC1367	CUP1p-MTS-less-MMF1-Flag-MS2bps-URA3	This work
pMAC1424	CUP1p-Mmf1MTS-COX4-Flag-ADH1t-URA3	This work
pMAC1425	CUP1p-MMF1 $_{AAA92AAG,\ GTA95GTC}$-Flag-ADH1t-URA3	This work
pMAC1430	CUP1p-MMF1-Flag-MS2bps-ura3::HIS3	This work
pMAC1431	CUP1p-MTS-less-MMF1-Flag-MS2bps-ura3::HIS3	This work
pE23	ura3::HIS3	pUH7 Daignan Fournier lab, Cross 1997
pE24	ura3::LEU2	pUL9 Daignan Fournier lab, Cross 1997
pE116	pRS315	Euroscarf
pE298	CUP1p-UBI-6His	Dargemont lab
pE617	CUP1p-COX12-Flag	Agnes Chacinska lab
pE659	pET264-puc24xMS2V6-U-Var-LoxP_KanMX4_LoxP	Evelina Tutucci lab

Internal number	Oligo description	Sequence
687	MMF1 qPCR for	CAACGTATTCTTGGCTGACATG
691	Cox4 For EcoRI	AAAAAAGAATTCATGCTTTCACTACGTCAATCTATAAGATTTTTCAAGCC
692	Cox4 Rev Not1	ACTAGTGCGGCCGCGTGATGGTGGTCATCATTTGGAACACC
714	COX4 qPCR for	CCGTTCCAACAGACCTAGATCAAG
935	MMF1 Rv Not1	TTT TTT GCG GCCGCA TTC TTT TCA ACA GCG ATA ACT TCC
936	MMF1 MfeI Fw	AAA AAA CAA TTG ATG TTT TTA AGA AAT TCC GTT TTG AG
999	Flag R	CTTATCGTCGTCATCCTTGATATC
1000	EGD2 F	AAGACGTCGCTACCAAGTCC
1001	EGD2 R	GTCACCGGCATCAACTTCAC
1009	MMF1 without target sequence	CGAAATAGCAATTGATGATAACAACATTGACCCCGGTCAG
1028	MMF1 without target sequence	CTTGTTAGTGCAATATCATATAGAAGTCATCGAAATAG GAATTCATGATAACAACATTGACCCCGGTCAG
1029	MMF1 with COX4 target sequence	CTTGTTAGTGCAATATCATATAGAAGTCATCGAAATAG GAATTCATGCTTTCACTACGTCAATCTATAAGATTTTTCAAGCCAGCCACAAGAACTTTGTGTAGCTCTAGAATAACAACATTGACCCCGGT
1030	MMF1 reverse Not1	CTTATCGTCGTCATCCTTGTAATCCATCGATACTAGTGCGGCCGCATTCTTTTCAACAGCGATAACTTCC
1087	MMF1 F	TATCGCTGTTGAAAAGAATGCGGCCGCACTAGTATCGATGGATTACAAGGATGACGACGATAAGATCTGACCGCTCTAGAACTAGTGGAT
1088	MMF1 R	GTAGACAAGCCGACAACCTTGATTGGAGACTTGACCAAACCTCTGGCGAAGAATTGTTAATTAAGAGCTCATTAAGGGTTGTCGAGATCG
1113	MS2 R	GAGAAAGCAACCTGACCTACAGGA
1297	MTS-Mmf1-Cox4 F	ACAGCTCCAGTCTTGAGGAGGGGTATATATCTGCTTCAGCAAAAACC
1298	MTS-Mmf1-Cox4 R	TCTCAAAACGGAATTTCTTAAAAACATGAATTGATACAAGACAAGGA
1301	Change Mmf1 codons 92, 95 F	TTAGACAATATAGTCAAGGTCAACGTCTTCTTGGCTGACATG
1302	Change Mmf1 codons 92, 95 R	AGAAGAATTACTTTCTGCTAAGATATTCTTAACGTT

Internal Number	Strains	Genotype	Reference
1	WT	MAT a gcn4Δ ura3-52 trp1Δ1 leu2::PET56 gal2	KY803, Hope and Struhl, 1986
3415	WT	MAT α his3 leu2 ura3 lys2	Euroscarf
3417	not4Δ	MAT α his3 leu2 ura3 lys2 not4::KanMX	Euroscarf
3421	caf130Δ	MAT α his3 leu2 ura3 lys2 caf130::KanMX	Euroscarf
3422	ccr4Δ	MAT α his3 leu2 ura3 lys2 ccr4::KANMX	Euroscarf
3465	vms1Δ	MAT α his3 leu2 ura3 lys2 vms1::KanMX	Euroscarf
3612	Egd1-HA3	MAT a gcn4Δ ura3-52 trp1Δ1 leu2::PET56 gal2 egd1::EGD1-3HA-KanMX4	Derived from KY803, Panasenko et al., 2006
4555	caf130Δ Egd1-HA3	MAT α ura3 leu2 his3 trp1 caf130::KanMX4 egd1::EGD1-HA3-KanMX4	Euroscarf
8662	atg17Δ	MAT a leu2Δ0 ura3Δ0 met15Δ0 his3Δ1 atg17::KanMX4	Euroscarf
10005	pre1-1	pre1-1	From Mafalda Escobar-Henriques lab
10451	ssaΔ	ura3-52 leu2-3,112 his3-11 lys2 trp1Δ1 ssa1-45 ssa2::LEU2 ssa3::TRP1 ssa4::LYS2 prc1-1 (CPY*)	From Wolf Dieter lab
10652	NAC Δ	his3 trp1 leu2 ura3 egd1::URA3 egd2::ADE2 btt1::HIS3MX4	From Sabine Rospert lab
11809	hel2Δ	MAT a his3Δ1 leu2Δ0 met15Δ0 ura3Δ0 hel2::kanMX4	Euroscarf
12303	RPS7A-K4R	MAT a leu2-3,112 trp1-1 can1-100 ura3-1 ade2-1 his3-11,15 rps7a::HISMX4 rps7b::NATMX4 pLEU2-rps7a-4KR-HA	W303-1a and PCR from Toshi Inada pRS315 based plasmid (K72, K76, K83 and K84)
12304	RPS7A	MAT a leu2-3,112 trp1-1 can1-100 ura3-1 ade2-1 his3-11,15 rps7a::HISMX4 rps7b::NATMX4 pLEU2-RPS7A-HA	W303-1a and PCR from Toshi Inada pRS315 based plasmid
12971	atg32Δ	MAT a leu2Δ20 ura3Δ met15Δ his3Δ1 atg32::URA3	KOY1380 (BY4741 background)
13549	Mmf1-Taptag	MATa ade2 arg4 leu2,3112 trp1-289 ura3-52 mmf1::MMF1-Taptag-URA3	Euroscarf
13551	msp1Δ	MAT α his3 leu2 ura3 lys2 msp1::KANMX4	Euroscarf
13701	Mmf1-Taptag not4 Δ	MATα his3Δ leu2Δ lys2Δ0 ura3Δ not4::NATMX4 mmf1::MMF1-Taptag-URA3	This work
13784	egd1Δ	MAT α his3 leu2 ura3 egd1::KANMX4	Euroscarf
13785	hsp104Δ	MAT α his3 leu2 ura3 lys2 hsp104::KANMX4	Euroscarf
13787	om45Δ	MAT α his3 leu2 ura3 lys2 om45::KANMX4	Euroscarf
13790	om14Δ	MAT α his3 leu2 ura3 lys2 om14::KANMX4	Euroscarf
13791	cis1Δ	MAT α his3 leu2 ura3 lys2 cis1::KANMX4	Euroscarf
13989	tom20Δ	tom20::LEU2	SNY1005, from Shuh-ichi Nishikawa, W303-1A background
14470	atg11 Δ	MAT α his3 leu2 ura3 lys2 atg11::KanMX	From Mafalda Escobar-Henriques lab
14471	atg5 Δ	MAT α his3 leu2 ura3 lys2 atg5::KanMX	From Mafalda Escobar-Henriques lab
14472	atg8 Δ	MAT α his3 leu2 ura3 lys2 atg8::KanMX	From Mafalda Escobar-Henriques lab
14486	sam37 Δ	MAT α his3 leu2 ura3 lys2sam37::KanMX	Euroscarf
14492	ski2Δ	MAT α his3 leu2 ura3 lys2	From Rachel Green lab
14493	xrn1Δ	MAT α his3 leu2 ura3 lys2	From Rachel Green lab
14612	Su9-mCherry	MATa trp1::Su9-mCherry-TRP1 ade2 leu2 his3 ura3 can1 SUC2	From Brian Zid lab ZY783
14613	su9-mCherry MCP-4GFP	MATa trp1::Su9-mCherry-TRP1 ade2 leu2 his3 ura3::CYC1p-MCP-4GFP-URA3 can1 SUC2	From Brian Zid lab ZY801

	Antibodies		Reference
Commercial	PAP（Peroxidase Anti Peroxidase complex）		Sigma （P1291）
Commercial	Flag	Monoclonal	Sigma （F3165）
Homemade	Por1	Polyclonal	From Yoav Arava lab
Homemade	Hxk1	Polyclonal	From Yoav Arava lab
Commercial	HA	Monoclonal	Sigma （H3663）
Homemade	Egd2	Polyclonal	Panasenko et al., 2009

References

Panasenko, O., Landrieux, E., Feuermann, M., Finka, A., Paquet, N. and Collart, M.A. (2006) The yeast Ccr4-Not complex controls ubiquitination of the nascent-associated polypeptide (NAC-EGD) complex. *J Biol Chem* , **281**, 31389-31398.

Panasenko, O.O., David, F.P. and Collart, M.A. (2009) Ribosome association and stability of the nascent polypeptide-associated complex is dependent upon its own ubiquitination. *Genetics* , **181**, 447-460.

Panasenko, O.O. and Collart, M.A. (2012) Presence of Not5 and ubiquitinated Rps7A in polysome fractions depends upon the Not4 E3 ligase. *Mol Microbiol* , **83**, 640-653.

Cross, F.R. (1997) 'Marker swap' plasmids: convenient tools for budding yeast molecular genetics. *Yeast* , **13**, 647-653.

Hope, I.A. and Struhl, K. (1986) Functional dissection of a eukaryotic transcriptional activator protein, GCN4 of yeast. *Cell* , **46**, 885-894.
